# Retinoic Acid Metabolic Genes, Meiosis, and Gonadal Sex Differentiation in Zebrafish

**DOI:** 10.1371/journal.pone.0073951

**Published:** 2013-09-10

**Authors:** Adriana Rodríguez-Marí, Cristian Cañestro, Ruth A. BreMiller, Julian M. Catchen, Yi-Lin Yan, John H. Postlethwait

**Affiliations:** 1 Institute of Neuroscience, University of Oregon, Eugene, Oregon, United States of America; 2 Departament de Genètica, Universitat de Barcelona, Barcelona, Spain; Temasek Life Sciences Laboratory, Singapore

## Abstract

To help understand the elusive mechanisms of zebrafish sex determination, we studied the genetic machinery regulating production and breakdown of retinoic acid (RA) during the onset of meiosis in gonadogenesis. Results uncovered unexpected mechanistic differences between zebrafish and mammals. Conserved synteny and expression analyses revealed that *cyp26a1* in zebrafish and its paralog *Cyp26b1* in tetrapods independently became the primary genes encoding enzymes available for gonadal RA-degradation, showing lineage-specific subfunctionalization of vertebrate genome duplication (VGD) paralogs. Experiments showed that zebrafish express *aldh1a2*, which encodes an RA-synthesizing enzyme, in the gonad rather than in the mesonephros as in mouse. Germ cells in bipotential gonads of all zebrafish analyzed were labeled by the early meiotic marker *sycp3*, suggesting that in zebrafish, the onset of meiosis is not sexually dimorphic as it is in mouse and is independent of Stra8, which is required in mouse but was lost in teleosts. Analysis of *dead-end* knockdown zebrafish depleted of germ cells revealed the germ cell-independent onset and maintenance of gonadal *aldh1a2* and *cyp26a1* expression. After meiosis initiated, somatic cell expression of *cyp26a1* became sexually dimorphic: up-regulated in testes but not ovaries. Meiotic germ cells expressing the synaptonemal complex gene *sycp3* occupied islands of somatic cells that lacked *cyp26a1* expression, as predicted by the hypothesis that Cyp26a1 acts as a meiosis-inhibiting factor. Consistent with this hypothesis, females up-regulated *cyp26a1* in oocytes that entered prophase-I meiotic arrest, and down-regulated *cyp26a1* in oocytes resuming meiosis. Co-expression of *cyp26a1* and the pluripotent germ cell stem cell marker *pou5f1*(*oct4*) in meiotically arrested oocytes was consistent with roles in mouse to promote germ cell survival and to prevent apoptosis, mechanisms that are central for tipping the sexual fate of gonads towards the female pathway in zebrafish.

## Introduction

A critical stage in vertebrate sex determination is entry of the bipotential gonadal primordium into a developmental pathway leading to ovary or testis (reviewed in 1). Sex-specific programs depend upon cell signaling between developing germ cells and somatic cells. Discovery of Sry (sex-determining region Y), the major sex-determining gene in mammals [2,3], stimulated the search for genetic mechanisms that control the sexual fate of somatic and germ cells during vertebrate gonadogenesis. In some vertebrates, however, such as zebrafish, developmental genetic mechanisms that control sexual fate remain unknown [4–9], and the identification of sex-biasing genes has been elusive [10,11]. For a zebrafish stock recently derived from India, a major sex determinant was identified on a chromosome that has several features of a sex chromosome [12], but genetic analyses of several laboratory strains identified different polygenic factors in different strains [12–15].

In mammals, male and female germ cells enter meiosis at different developmental stages (reviewed in 16–19). In female embryonic mouse gonads, germ cells enter into meiosis at 13.5 days post-coitum (dpc) and concomitantly, the somatic gonadal primordium initiates ovarian differentiation by developing into granulosa and theca cells. In contrast, in male embryonic mouse gonads, SRY-expressing pre-Sertoli cells initiate testicular differentiation and germ cells arrest in the G0/G1 phase of the mitotic cell cycle, postponing meiosis until after birth [20,21]. Furthermore, meiotic XX germ cells antagonize testicular development in XY gonads in tissue co-culture experiments, leading to the hypothesis that germ cells committed to meiosis reinforce ovarian fate by antagonizing the testis pathway [22].

Recent studies showed that in mouse, retinoic acid (RA) in females or its absence in males -- regulated by the RA-destroying Cyp26 Cytochrome P450 enzyme family -- reinforces germ cell fate by controlling the sex-specific initiation of meiosis ( [23–27] reviewed in 18,28; although see 29 for an alternative hypothesis). Retinoic acid is an active derivative of vitamin A that diffuses through tissues and binds to heterodimers of the nuclear receptors RAR-RXR, which recognize RA-response elements (RAREs) in DNA to control the expression of RA-target genes [30–33]. Because *Rar* and *Rxr* are widely expressed in embryos, the regulation of RA action often occurs at the metabolic level, in which the balance of RA-synthesizing enzymes (i.e. the Aldh1a retinaldehyde dehydrogenase family) and RA-degrading enzymes (i.e. members of the Cyp26 P450-cytochrome family) determines the precise spatial-temporal distribution of RA [34]. This is the case in mouse gonadogenesis, in which male-specific up-regulation of *Cyp26b1* expression leads to degradation of RA and protects germ cells from entering into meiosis in developing testes, while female-specific down-regulation of *Cyp26b1* expression allows RA to induce germ cells to enter into meiosis in embryonic ovaries [23,24,35]. The central role of Cyp26b1 in preventing entry into meiosis was further supported by evidence showing that disruption of *Cyp26b1* expression in embryonic mouse testes, or addition of CYP inhibitors to wild-type embryonic testes, induced germ cells to express the pre-meiotic marker *Stra8* (*stimulated by retinoic acid-8*), which is required for the initiation of meiosis in mammals, followed by the expression of the early meiotic marker *Sycp3* (*synaptonemal complex protein 3*), and the down-regulation of the pluripotent stem cell marker *Pou5f1* (*POU* class *5 homeobox 1*, or *Oct4*) [23,24,35–45].

In mice, expression of the RA-synthesizing enzyme Aldh1a2 in the embryonic mesonephros (but not in the gonads) as females initiate meiosis led to the suggestion of a source-sink regulatory system. According to this model, RA synthesized in the mesonephros enters the neighboring gonad and causes germ cells to enter meiosis in embryonic ovaries, which lack the RA-degrading enzyme Cyp26 ( [23,24,42]; reviewed in 18). Recently, male-specific expression of the *Aldh1a2* paralog *Aldh1a1* has been reported in somatic cells of embryonic mouse testes [46]. The expression of Aldh1a1, however, has been suggested to act as a buffer to maintain low levels of RA that might be needed for general testis morphogenesis rather than the high levels of RA needed for germ cells to enter meiosis [46].

RA plays a role in the onset of meiosis not only in mammals, but also in other tetrapods, including birds and amphibians [47–49]. Whether the role of RA during gonad development is a tetrapod innovation, however, or whether it is shared with other non-tetrapod vertebrates, including teleost fishes, remains unknown [50]. Similarities and differences in the mechanisms of gonadogenesis in teleosts and tetrapods suggest the question: Is RA action important for meiosis and gonadogenesis in zebrafish? Several considerations motivate this problem. First, in contrast to mouse, the gonads of a zebrafish do not lie adjacent to the mesonephros during the critical period for gonadal sex determination; consequently the source-sink regulatory system from the mesonephros to the gonad postulated in mouse is unlikely to apply to zebrafish. Second, in contrast to mouse, all zebrafish juveniles, regardless of their definitive sex, initially develop an ovary-like gonad with immature oocytes; in females, these oocytes continue to develop and reinforce the differentiation of mature ovaries, but in males, oocytes die by apoptosis and the gonads become testes [4,5,7,8,51–53]. We do not yet know, however, whether differences in the timing of the onset of meiosis exist between zebrafish males and females. Third, genomic surveys of the RA-metabolic genetic machinery have shown that some *aldh1a* family genes (i.e. *aldh1a1*) have been lost in zebrafish and other teleosts, and that this gene loss has altered the functional evolution of the surviving *aldh1a* paralogs [54–57]. Whether these gene losses had functional consequences in gonad development is not yet known.

To address these questions and to test the hypothesis that meiotic control mechanisms in mouse apply to zebrafish, we performed a comprehensive genomic and expression analysis of the genetic machinery that regulates the synthesis and degradation of RA during gonadogenesis, and studied genetic markers for meiosis and somatic cells of the gonad to investigate the role of RA in the onset of meiosis and sexual fate determination in zebrafish. Results revealed shared underlying regulatory themes between zebrafish and mammals but important genomic and developmental differences in the mechanisms of RA-regulated gonadogenesis and sex determination.

## Results

### 1. In zebrafish, *cyp26a1*, rather than *Cyp26b1* as in mammals, is the main RA-degradation gene expressed during gonad development

Tetrapods and zebrafish have three *Cyp26* paralogs: *Cyp26a1, Cyp26b1*, and *Cyp26c1*. Recent studies in mouse [23,24], chicken [47,49] and urodele amphibians [48] implicated the RA-degrading enzyme Cyp26b1 as the pivotal sex-specific factor regulating the differential timing of meiotic onset in females and males. Our first question was whether male-specific up-regulation of *cyp26b1* occurs during gonad development in zebrafish as it does in tetrapods.

**Figure 1:**
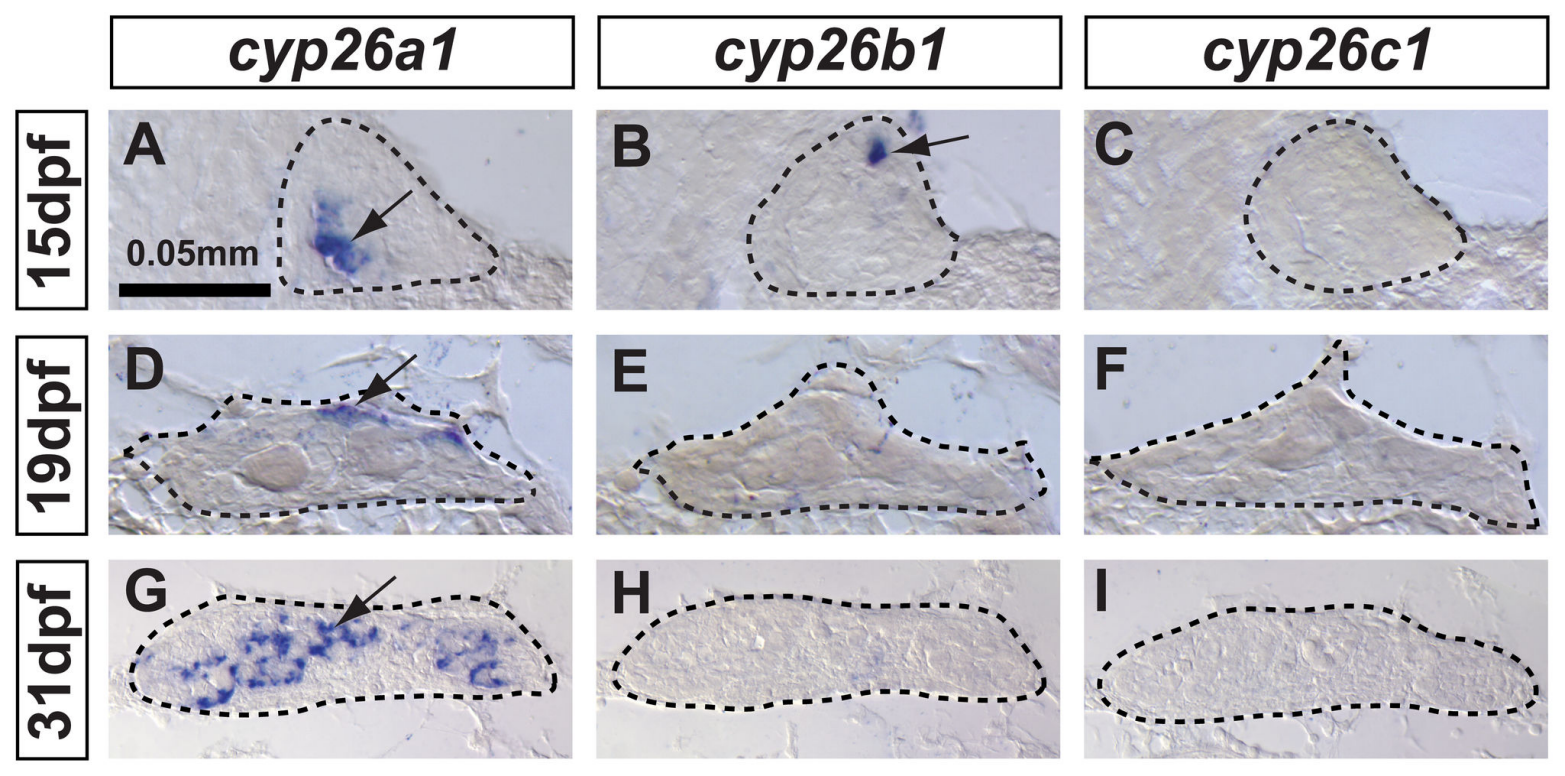
In zebrafish, *cyp26a1* is the main *Cyp26* paralog expressed in gonads during the critical period for sex-determination. Undifferentiated gonads at 15 days post-fertilization (dpf) expressed *cyp26a1* (A) and *cyp26b1* (B), but did not express *cyp26c1* at detectable levels (C) (A-C: n=4). In bipotential gonads at 19 dpf, *cyp26a1* expression became restricted mainly to the dorsal margin of the gonad (D) but expression of *cyp26b1* and *cyp26c1* was not detected (E, F) (D–F: n=9). In differentiating testes at 31 dpf, *cyp26a1* expression up-regulated (G) and in contrast to mouse testes, which up-regulate *cyp26b1*, neither *cyp26b1* nor *cyp26c1* expression was detected in maturing zebrafish gonads (H, I) (G-I: n=10). Differentiating testes were assigned by morphological features and assessed by the expression of the male specific *Amh* marker (see Figure 4). We conclude that in zebrafish, Cyp26a1 is expressed at the time and place necessary to provide an RA-degrading function equivalent to Cyp26b1 in tetrapods. These results suggest independent subfunction partitioning of ancestral *cyp26* regulatory elements in lineages leading to zebrafish and mouse. Arrows point to examples of expressing cells. Dashed lines outline gonads. Scale bar: 0.05mm.

To address this question, we investigated expression patterns of c*yp26b1*, as well as the other two gene family members *cyp26a1* and *cyp26c1*, in adjacent histological sections of developing zebrafish gonads by *in situ* hybridization (ISH) (Figure 1A–I). Zebrafish *cyp26a1* expression was detected in several cells in the gonadal primordium at 15 dpf before the critical period for sex determination (Figure 1A), became more abundant and mostly restricted to the dorsal surface of the gonad close to the body cavity by 19 dpf during early sex differentiation (Figure 1D), and was highly up-regulated and broadly expanded by 31 dpf during the immature testis stage (Figure 1G). This result revealed that *cyp26a1* was expressed in zebrafish gonads early and continuously through the critical time window for gonadal sex determination (19 dpf - 31dpf) [4,5,8,58–60]. Unexpectedly, expression of zebrafish *cyp26b1* was detected only in a few scattered cells in undifferentiated gonads at 15 dpf (Figure 1B), and in contrast to its ortholog in tetrapods, *cyp26b1* was not detected in gonads of animals at the stages in which sex determination occurs (19 dpf -31 dpf, Figure 1E,H). No *cyp26c1* expression was detected in developing gonads at any stage analyzed (Figure 1C,F,I), although positive controls did show *cyp26c1* expression in the hindbrain of zebrafish embryos (data not shown) and in the retina of 15 dpf juveniles. These results revealed that, in contrast to tetrapods, in which *cyp26b1* is the major regulator of RA availability during early gonad development, *cyp26a1* is the only paralog up-regulated in developing zebrafish gonads during the time of sexual differentiation of the gonads.

### 2. Genomic synteny conservation and independent subfunction evolution of the Cyp26 family in teleosts and tetrapods

**Figure 2:**
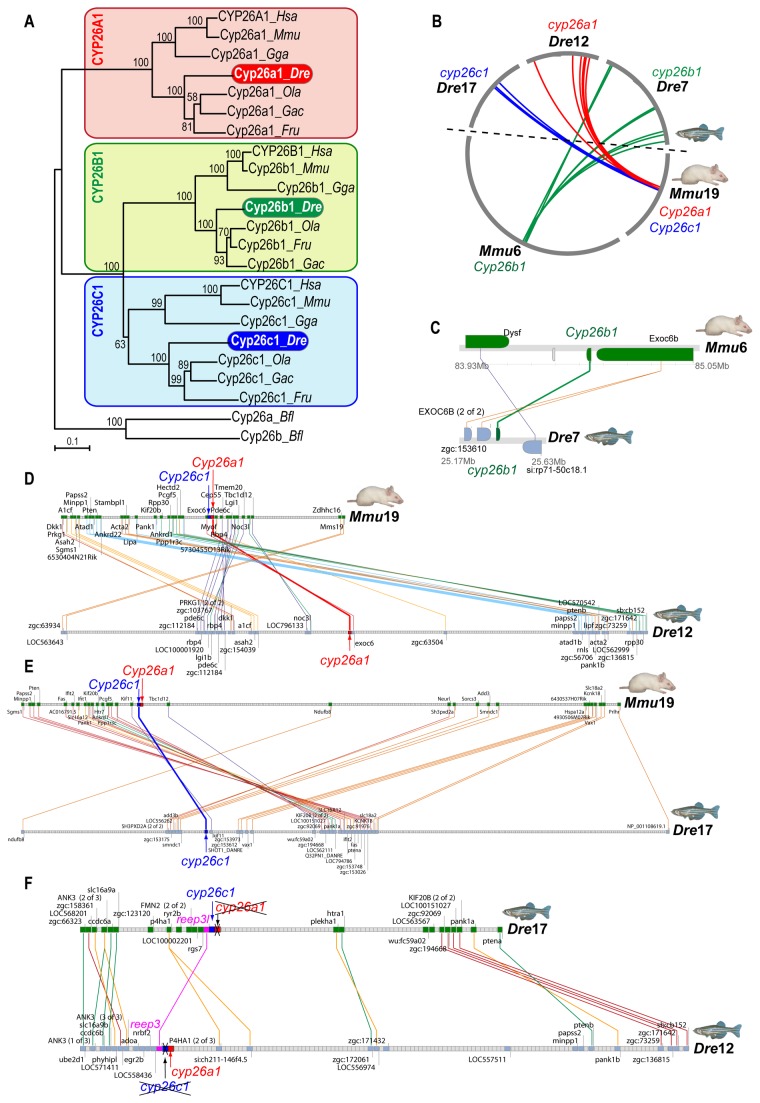
Evolutionary relationships of CYP26 family members in zebrafish and mouse. (A) Phylogenetic analyses inferred by maximum likelihood (ML) indicate that Cyp26 paralogs of teleosts (i.e. zebrafish (Dre), medaka (Ola), stickleback (Gac) and fugu (Tru)) grouped with their correspondingly named Cyp26 paralogs of tetrapods (i.e. mouse (Mmu), human (Hsa) and chicken (Gga)). Numbers indicate bootstrap values supporting each node (n=100), and no significant differences were found between ML and NJ analyses. (B) A circleplot shows graphically orthologous relationships of *cyp26* genomic neighborhoods shared between zebrafish and mouse. Grey circumferential arcs represent chromosomes, with *Cyp26b1* in green on *Mmu*6, *Cyp26a1* in red on Mmu19, and *Cyp26c1* in blue on *Mmu*19. Colored lines link orthologous regions in zebrafish chromosomes *Dre*7, *Dre*12 and *Dre*17, the sites of *cyp26b1*, *cyp26a1* and *cyp26c1* genes, respectively. (C–E) Clusters of synteny conservation reveal the presence of many gene neighbor orthologs shared between each *Cyp26* genomic neighborhood in mouse and zebrafish *cyp26b1* (C: cluster ID#265419 according to the Synteny Database), *cyp26a1* (D: cluster ID#258723) and *cyp26c1* (E: cluster ID#265367). These results rule out the possibility of reciprocal gene losses in zebrafish and mouse that could mask actual orthologous relationships in artifactual phylogenetic trees, and provide strong support for the conclusion that the zebrafish/mouse gene pairs called *cyp26a1/Cyp26a1* and *cyp26b1/Cyp26b1* are in fact orthologs. F: Gene clusters of synteny conservation (cluster ID#191383) between *Dre12* and *Dre17* reveal that the genomic neighborhoods of *cyp26a1* and *cyp26c1* are paralogous due to the teleost genome duplication (TGD, R3) that preceded the divergence of the teleost lineage after it split from the tetrapod branch. Duplicates of *cyp26a1* and *cyp26c1* in *Dre*17 and *Dre*12 were lost reciprocally after the teleost genome duplication (labeled with crosses) in contrast to, for instance, *reep3* paralogs (in pink) that were maintained adjacent to *cyp26* paralogs.

The apparent convergence of the roles of Cyp26a1 in zebrafish and Cyp26b1 in mouse could have either of two explanations: first, it might represent a case of independent subfunctionalization of gene roles possessed by the ancestral *Cyp26* gene before the two rounds of vertebrate genome duplications (alias VGD1 and VGD2); alternatively, it might be that zebrafish gene orthologies had been incorrectly assigned. To rule out the possibility of error in orthology assignments, we examined gene orthologies by first investigating the phylogenetic relationships between zebrafish Cyp26a1 [61], Cyp26b1 [62] and Cyp26c1 [63] (originally called Cyp26d1 [64]) and their respective mouse paralogs [65–67]. Phylogenetic analysis by Maximum-likelihood showed that the three Cyp26 paralogs of various teleosts (zebrafish, medaka, stickleback and fugu) grouped within the clades of their correspondingly named tetrapod Cyp26 paralogs with high bootstrap support (Figure 2A), corroborating the recently proposed Cyp26 nomenclature [63].

Phylogenetic analyses can sometimes give erroneous orthology assignments in cases of reciprocal gene loss after ancient gene duplications [68–70]; for example, four *Cyp26* genes would have been produced in VGD1 and VGD2 [71], but zebrafish and tetrapods both have three rather than four *Cyp26* genes, raising the possibility that different VGD paralogs were lost in the teleost and tetrapod lineages.

To critically test the hypothesis that reciprocal gene loss occurred in the Cyp26 family in the zebrafish and mouse lineages leading to erroneous orthology assignments, we examined a data set independent of Cyp26 amino acid sequence by examining syntenic conservation within the genomic neighborhoods (GN) surrounding *Cyp26* genes in zebrafish and mouse (Figure 2B–F) and medaka (Figure S1, Figure S2). In mouse, the three *Cyp26* paralogs reside on two chromosomes: *Cyp26b1* is on mouse chromosome 6 (Mmu6), and *Cyp26a1* and *Cyp26c1* are adjacent and transcribed in the same orientation on Mmu19, consistent with the origin of *Cyp26a1* and *Cyp26c1* by tandem duplication rather than by genome duplication. In teleosts, however, the three Cyp26 paralogs are on three different chromosomes (i.e. *cyp26a1* on zebrafish (*Danio rerio*) linkage group 12 (Dre12) and on medaka (*Oryzias latipes*) chromosome 19 (Ola19); *cyp26b1* on Dre7 and Ola18; and *cyp26c1* on Dre17 and Ola15). Genomic analysis at the chromosomal level using circleplots [69] revealed orthology relationships between each Cyp26 chromosomal neighborhood in mouse with its corresponding orthologous chromosomal region in zebrafish (Figure 2B) and medaka (Figure S2A): The mouse *Cyp26b1* genomic neighborhood connected to teleost *cyp26b1* neighborhoods; and the mouse *Cyp26a1*/*Cyp26c1* neighborhood connected to teleost neighborhoods that harbor *cyp26a* or *cyp26c1*, respectively (Figure 2B and Figure S2A). Local analysis of each Cyp26 genomic neighborhood using the Synteny Database (version Ens56 [69]) identified conserved gene neighbors near each Cyp26 ortholog: *Cyp26b1* with *Dysf* (Figure 2C and Figure S2B), *cyp26a1* with *Pde6c* (Figure 2D and Figure S2C), and *cyp26c1* with *Tbc1d12* (Figure 2E and Figure S2D). These results provide robust evidence that rules out the hypothesis that reciprocal Cyp26 paralog loss occurred between zebrafish and mouse.

Comparative analysis between zebrafish genomic neighborhoods surrounding *cyp26a1* in *Dre*12 and *cyp26c1* in *Dre*17 revealed that these two regions are indeed paralogons (Figure 2F). This finding suggests the hypothesis that prior to the tetrapod-teleost divergence, *cyp26a1* and *cyp26c1* were already adjacent in an ancestral chromosome due to a tandem duplication, and that after the teleost genome duplication (TGD) event (reviewed in 72), *cyp26a1* and *cyp26c1* duplicates survived reciprocally in each paralogon, leading to the present situation in which *cyp26a1* and *cyp26c1* are on different teleost chromosomes (Figure 2F and Figure S2E).

Overall, our phylogenetic and comparative genomic analyses provided robust evidence that rules out the hypothesis of reciprocal *Cyp26* gene losses in the zebrafish and mouse lineages and supports the recently postulated Cyp26 ontogeny [63]. We conclude, therefore, that the most parsimonious explanation is that the *Cyp26* pro-ortholog predating the expansion of the gene family was already involved in the regulation of RA levels during gonad development, and that both Cyp26a1 and Cyp26b1 maintained this function in the last common ancestor of zebrafish and mouse. Subsequently, independent subfunction partitioning [72,73] likely led to the reciprocal retention of the gonad function of Cyp26a1 in the lineage leading to zebrafish and Cyp26b1 in the tetrapod lineage.

**Figure 3:**
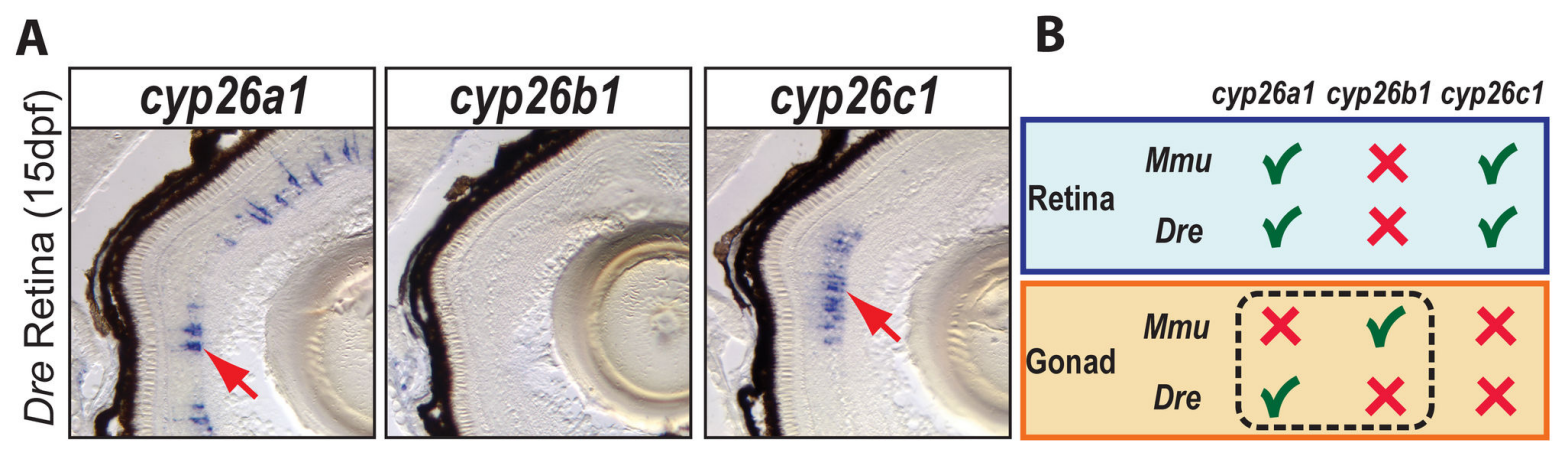
Expression patterns of *cyp26* gene family members in late developing retina are conserved between zebrafish and mouse. To learn if the divergent subfunction partitioning between zebrafish *cyp26a1* and mouse *cyp26b1* in gonad development applies to other organs, we studied the expression of *cyp26* paralogs during retina development in 15-dpf zebrafish. Results revealed expression of *cyp26a1* and *cyp26c1* in different layers of the retina, but no detectable expression of *cyp26b1* (A: n=1). This result agrees with the *Cyp26* expression profile described in mouse retina (B), in which *Cyp26a1* and *Cyp26c1* genes were expressed in the inner nuclear layer during post-natal eye development while *Cyp26b1* was not expressed in the eye [74]. We conclude that independent subfunction partitioning related to gonad development occurred after the teleost-tetrapod lineage divergence, while subfunctions related to at least one other organ, the retina, are still conserved between *Cyp26* orthologs in different vertebrate lineages (B).

We wondered whether independent partitioning of subfunctions of the ancestral *Cyp26* gene that were unrelated to the gonad occurred in the same manner as subfunction partitioning in the gonad. Comparative analysis of the three *cyp26* paralogs during late eye development in zebrafish (Figure 3A) and mouse [74] revealed that the zebrafish ortholog of each of these three genes has the same expression pattern in the retina as its mouse ortholog. This result suggests that at least some ancestral subfunctions (i.e. in the retina) partitioned the same way in the three *Cyp26* genes, perhaps before the divergence of zebrafish and mouse lineages, but the gonadal subfunction partitioned reciprocally in the two lineages after they diverged (Figure 3B).

### 3. Comparative expression analysis of genes encoding RA-metabolic machinery and somatic and germ cell markers during zebrafish gonadogenesis

**Figure 4:**
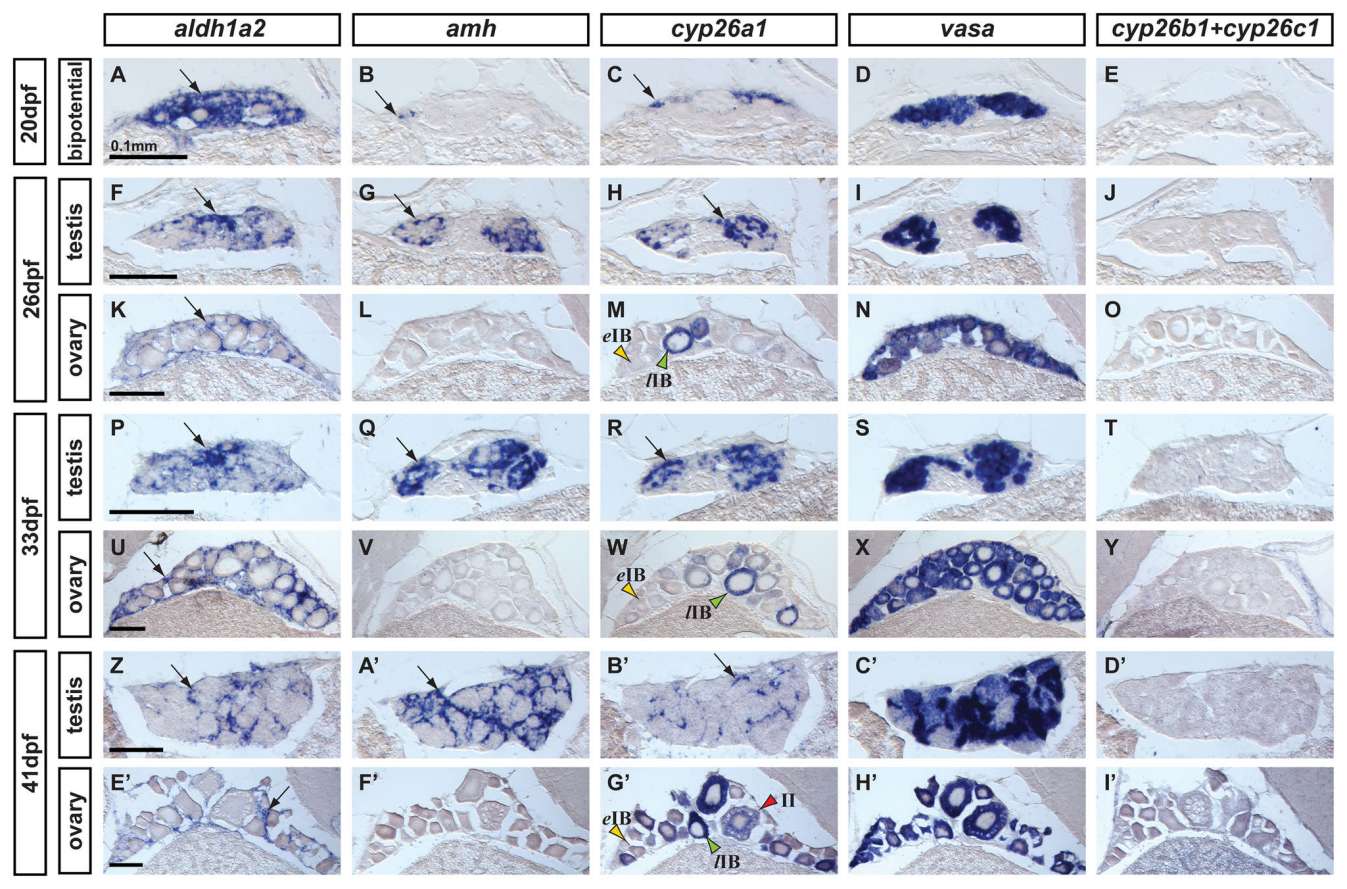
Expression of genes encoding enzymes for the synthesis and degradation of RA during zebrafish gonad development. *In situ* hybridization on adjacent sections of animals representing the three key stages of gonad development: (A–E: n=7) bipotential, sexually undifferentiated gonads with an ovary-like morphology at 20 days post-fertilization (dpf); (F–O) gonads transitioning to testes or ovaries (26 dpf) (F-J: n=4; K–O: n=3); (P–Y and Z–I’) gonads sexually differentiated but still immature (33 dpf and 41 dpf) (P–T: n=3; U–Y: n=4; Z-D’: n=2; E’–I’: n=2). Images show expression patterns of the gene encoding the RA-synthesizing enzyme Aldh1a2 (A, F, K, P, U, Z, E’), the gene encoding the RA-degrading enzyme Cyp26a1 (C, H, M, R, W, B’,G’) and combined probes for *cyp26b1* and *cyp26c1* (E, J, O, T, Y, D’, I’) together with the early male gonadal marker *amh* (*anti-Müllerian hormone*: B, G, L, Q, V & A’, F’) and the germ-line specific marker *vasa* (D, I, N, S, X, C’, H’) at four different stages. Expression of *aldh1a2* was detected in somatic cells in both male and female gonads throughout development (A, F, K, P, U, Z, E’). Expression of *aldh1a3* was not detected at all in gonads but was detected in retina cells (data not shown) and the ortholog of *Aldh1a1* was lost in the teleost lineage [54–56]. The expression pattern of *cyp26a1*, however, showed a distinct sexual dimorphism, as gonadal somatic cells from males (H, R and B’), but not from females (M, W, G’), up-regulated its expression during gonad development. Interestingly, in females, oocytes that had transitioned from early stage IB (yellow arrowhead in M,W,G’) to late stage IB (green arrowheads in M,W,G’) up-regulated the expression of *cyp26a1* in the ooplasm, which was maintained at later stages (e.g. red arrowhead stage II in G’). The observed expression pattern of *cyp26a1* in oocytes is compatible with a function in inhibiting meiotic progression and facilitating the meiotic arrest at diplotene stage. Expression of *cyp26b1* and *cyp26c1* in gonads was not detected at any of the stages analyzed in either sex (E, J, O, T, Y, D’, I’) Arrows point to examples of expressing cells. Scale bar shown per each row: 0.1mm.

To learn whether RA-producing and RA-degrading enzymes are sexually dimorphic in developing gonads, we compared male and female expression patterns of *cyp26* genes, which encode RA degrading enzymes, and the two zebrafish *aldh1a* paralogs (*aldh1a2*, and *aldh1a3*), which encode RA synthesizing enzymes. Because no robust genetic marker for sex is yet available for zebrafish [12,13], to assess sexual fate of zebrafish gonads, we examined expression of the early male-specific somatic marker amh (anti-Mullerian hormone) [59] and the germ cell-specific marker *vasa* [75]. We performed *in situ* hybridization analyses on adjacent gonadal sections from animals representing three key stages of gonad development: i) bipotential gonads (20 days post-fertilization (dpf)) when gonads are sexually undifferentiated and can become either ovary or testis (Figure 4 A–E); ii) transitional gonads (26 dpf), when gonads are in the process of transitioning from the juvenile ovary-like stage to enter the female or male pathway and differentiate into ovaries or testes (Figure 4F–O); and iii) differentiated but immature gonads (33 dpf and 41 dpf), after sexual fate selection but before gametes mature (Figure 4P–Y and Z–I’ respectively).

**Figure 5:**
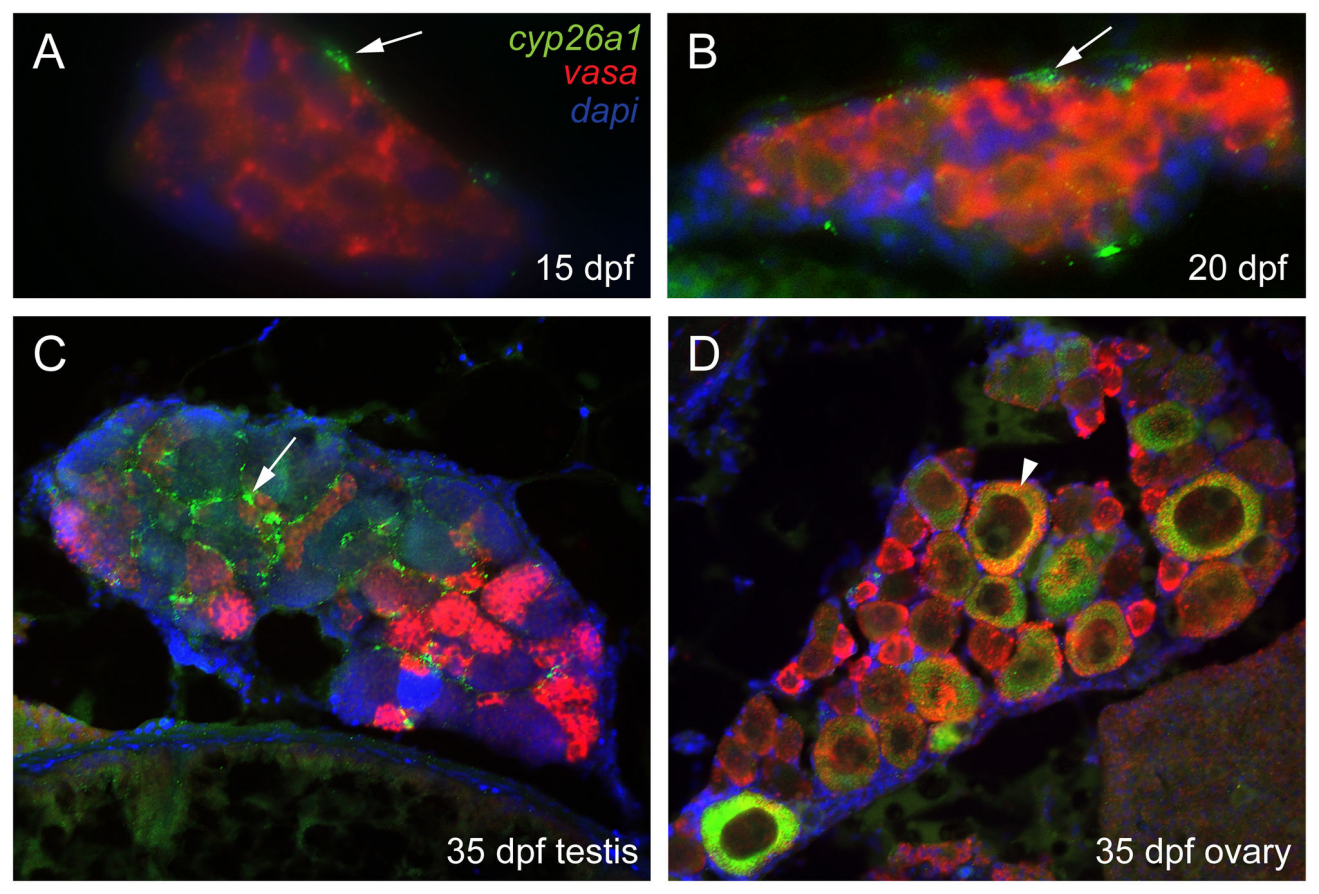
Three color fluorescent detection of *cyp26a1* and *vasa* expression during zebrafish gonad development. Fluorescence detection of *cyp26a1* (green) expression and *vasa* (red) expression by double *in situ* hybridization on gonad sections at the bipotential gonad stage at 15 dpf (A: n=1) and 20 dpf (B: n=2), and on immature gonads developing into testes (C: n=1) or ovaries (D: n=1) at 33 dpf. *Cyp26a1* expression occurs in somatic cells and does not co-localize with the germ cell marker *vasa* in bipotential gonads (A,B). In immature testes, *cyp26a1* expression was also expressed in somatic cells (arrow) and not in germ cells (C). In immature ovaries, however, *cyp26a1* was expressed in large oocytes that had reached diplotene (arrowhead in D).

In undifferentiated bipotential gonads at 20 dpf, *aldh1a2* was expressed throughout most cells of the ovary-like gonad of juveniles, but was not expressed in the largest germ cells, which were probably developing oocytes judging by their morphology (Figure 4A). No gonadal expression was observed for zebrafish *aldh1a3* (data not shown), which is the only other remaining *aldh1a* paralog in zebrafish due to the loss of *aldh1a1* early in the evolution of the teleost lineage [56]. In bipotential gonads, *amh* was already expressed, but in only a few scattered somatic cells (Figure 4B) [7,8,59]. At 20 dpf, *cyp26a1* maintained its expression at the surface of the gonad near the body cavity in all individuals (Figure 4C) as observed at 19 dpf (Figure 1D). Comparison of this thin superficial domain of *cyp26a1* expression to the broadly distributed domain *of vasa* expression in germ cells (Figure 4D) suggested that *cyp26a1* was expressed either in specific somatic cells or in a sub-set of germ cells (i.e. germ cell stem cells or gonia). Analysis by double fluorescent *in situ* hybridization in sections of bipotential gonads resolved this question, showing that *cyp26a1* was expressed in somatic cells but not in germ cells labeled by *vasa* (Figure 5A,B). No expression of *cyp26b1* or *cyp26c1* was detected in the gonad at this undifferentiated stage (Figure 4E).

Gonads transitioning from the juvenile ovary-like stage into definitive testis or ovary pathways (26dpf) showed broad expression of *aldh1a2* in gonads of both sexes (Figure 4F,K). Sex assignment was based on gonad morphology and the presence or absence of expression of the early male-specific somatic marker *amh* (Figure 4G,L). Comparison of the expression pattern of *aldh1a2* (Figure 4F,K) with the distribution of *vasa*-expressing germ cells (Figure 4I,N) suggested that the expression of *aldh1a2* was mostly restricted to somatic cells.

In transitional gonads, *cyp26a1* expression became strikingly sexually dimorphic. In males, expression of *cyp26a1* had extended from its prior thin superficial expression domain to include cells broadly distributed throughout the gonad (Figure 4H), likely somatic cells judged by the position of *vasa*-positive germ cells (Figure 4I). In contrast to males, females did not show detectable *cyp26a1* expression in somatic cells (Figure 4M). Instead, *cyp26a1* signal in females appeared restricted to the ooplasm of few large oocytes that had progressed to the diplotene stage of meiosis (i.e. late stage IB oocytes (*l*IB); green arrowhead in Figure 4M), but not in small, early stage oocytes (i.e. early stage IB oocytes (*e*IB); yellow arrowhead in Figure 4M). Because meiosis arrests at diplotene in late stage IB zebrafish oocytes – as occurs in human diplotene oocytes for as much as 40 or 50 years -- the up-regulation of *cyp26a1* in late stage IB is compatible with a potential role of Cyp26a1 in arresting oocytes during meiosis. Expression of *cyp26b1* and *cyp26c1* was not detected at this crucial stage for gonad fate decision in ovaries or testis (Figure 4J,O).

Gonads that had differentiated as immature ovaries or immature testes (33dpf and 41dpf) continued to express *aldh1a2* in somatic cells of gonads of both sexes (Figure 4P,U,Z,E’). In males, *aldh1a2* expression domain appeared to be restricted to a set of somatic cells surrounding testis cysts (Figure 4P,Z), judging from the broader expression of *amh* in developing Sertoli cells (Figure 4Q, A’). In females, *aldh1a2* expression continued to be restricted to somatic cells surrounding oocytes (Figure 4U,E’). These results suggest the continued potential for gonadal RA production in both sexes.

Differentiated but immature gonads continued to show a clear sexually dimorphic expression pattern for *cyp26a1* (Figure 4R,W,B’, G’), suggesting likely sex-specific patterns of RA degradation. In males, the broad *cyp26a1* expression domain observed at 33dpf (Figure 4R) became localized to a sub-set of somatic cells surrounding cysts of germ cells by 41dpf (Figure 4B’), judging by its more restricted distribution than that of *amh* (Figure 4A’) and its different distribution compared to *aldh1a2*-expressing cells (Figure 4Z). Expression of *cyp26a1* and *vasa* did not co-localize by double fluorescent *in situ* hybridization, corroborating the conclusion that *cyp26a1* was expressed in somatic but not germ cells in developing male gonads (Figure 5C). In 33dpf to 41dpf females, expression of *cyp26a1* continued to be restricted to the ooplasm of large oocytes that had reached diplotene (*l*IB green arrowhead in Figure 4W, G’ and white arrowhead in Figure 5D), while no *cyp26a1* expression was detected in the ooplasm of the smallest oocytes (*e*IB, yellow arrowhead in Figure 4W,G’ and Figure 5D). At 41-dpf, some oocytes had reached stage II (enlarged cells due to the formation of cortical alveoli) and consequently the *cyp26a1* expression signal became diluted (red arrowhead in Figure 4G’). No expression of *cyp26b1* and *cyp26c1* was detected in immature testes or ovaries at 31dpf and 41dpf (Figure 4T,Y,D’, I’).

### 4. *Somatic expression of aldh1a2 and cyp26a1 is independent of germ cell signaling*

**Figure 6:**
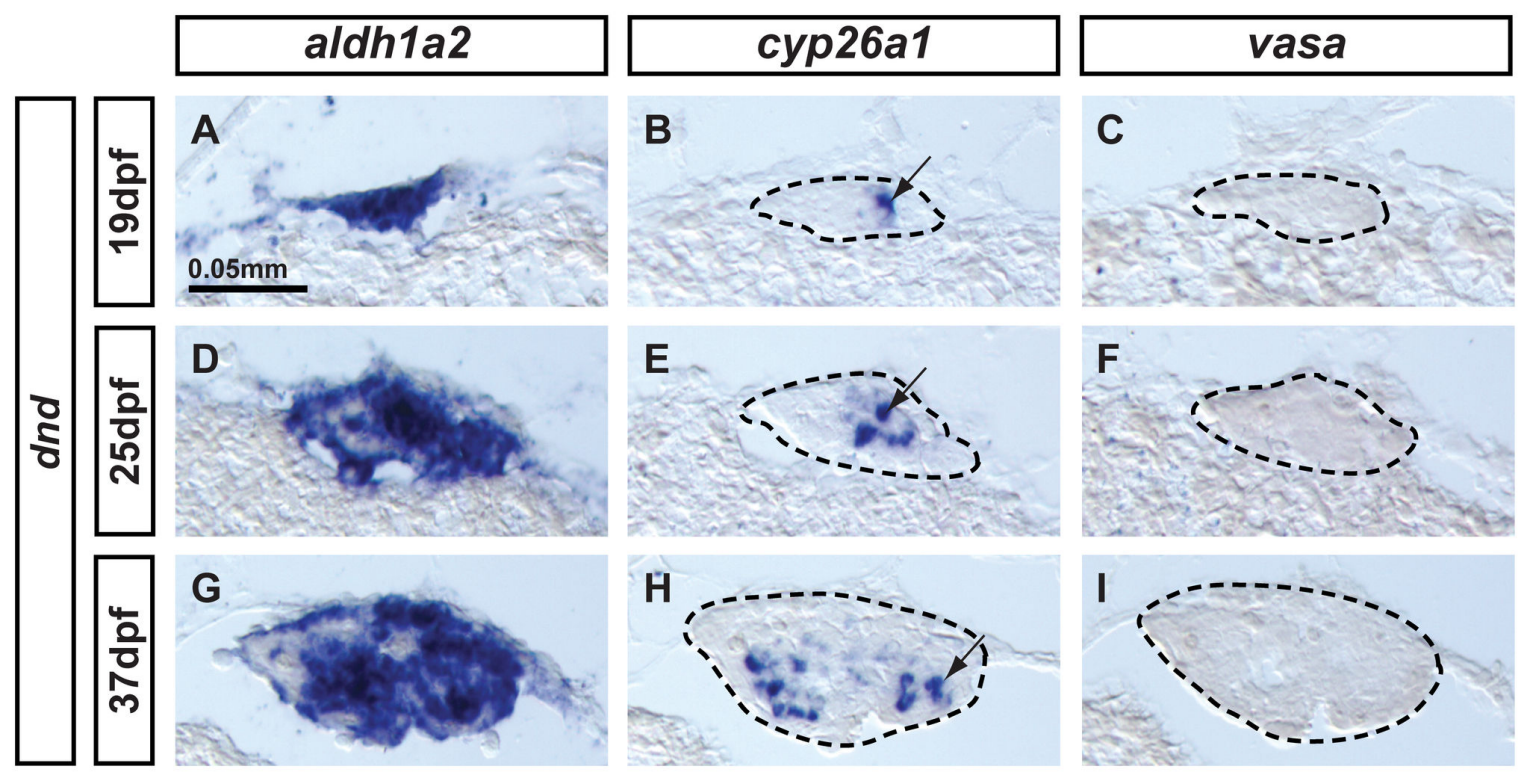
Expression of *aldh1a2* and *cyp26a1* is independent of germ cell signaling. Animals depleted of germ cells that develop into sterile males were generated by *dead end* (dnd) morpholino knockdown to study the expression of *aldh1a2* and *cyp26a1* in gonads at (A-C: n=1) bipotential stage (19dpf), (D–F: n=1) transitioning stage (25dpf), and (G-I: n=1) differentiated testes (37dpf). Results showed that *aldh1a2* was widely expressed in somatic cells of the gonads at the three different stages analyzed (A,D,G) while *cyp26a1* expression was detected in a subset of somatic cells also in the three stages analyzed (arrows in B, E, H). These results demonstrate that the onset as well as the maintenance of *aldh1a2* and *cyp26a1* expression, at least until 37dpf, is independent of germ cell signaling. Expression of the germ cell specific marker *vasa* was not detected at any stage (C,F,I), confirming the total depletion of germ cells by *dnd* morpholino injection in all animals. Gonads are outlined by a dashed line (B,C, E, F,H,I). Scale bar: 0.05 mm (A).

Germ cell signaling is essential for sex determination in zebrafish [6–8]. To test the hypothesis that germ cell signaling controls the somatic expression of *aldh1a2* and *cyp26a1*, we investigated the expression of both genes in knockdown zebrafish that had been depleted of germ cells by injection of antisense-morpholino (MO) against *dead end* (*dnd*), a gene essential for germ cell survival [76,77]. In situ hybridization experiments were performed on adjacent sections of gonads representing three different stages of gonad development: i) bipotential gonads at 19 dpf (Figure 6A–C); ii) transitioning gonads at 25dpf (Figure 6D–F); and iii) differentiated immature gonads at 37dpf (Figure 6G–I). The absence of expression of the germ cell specific marker *vasa* verified total depletion of germ cells in *dnd*-MO-injected animals (Figure 6C, F, I). Results revealed that *aldh1a2* (Figure 6A,D,G) and *cyp26a1* (Figure 6B,E,H) were both expressed in somatic cells despite the lack of germ cells at all stages examined. The *aldh1a2* expression domain spanned most of the gonad, except for a few islands that did not show signal, suggesting again the existence of a small subset of somatic cells that did not express *aldh1a2* as observed in male gonads (Figure 4F,P and Z). Expression of *cyp26a1* also occurred in the absence of germ cells, but in a smaller subset of somatic cells compared to the broader somatic expression domain of *aldh1a2*.

These experiments ruled out the hypothesis that the initiation or maintenance of *aldh1a2* and *cyp26a1* expression by somatic cells of the gonads depends on germ cell signaling in zebrafish at least until 37dpf.

### 5. Expression of RA-metabolic genes and somatic and germ-line markers in adult gonads

**Figure 7:**
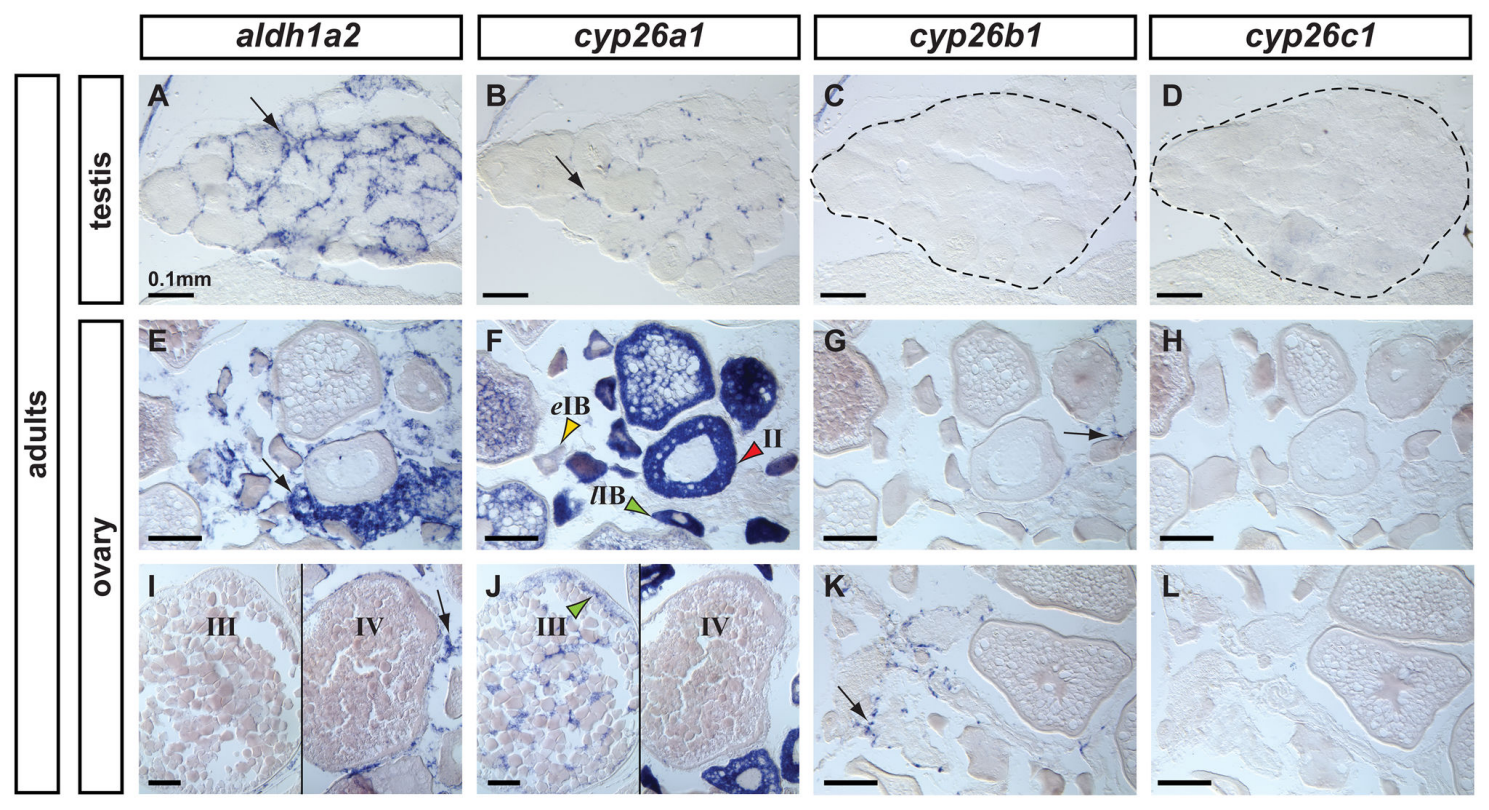
Expression patterns of *aldh1a2*, *cyp26a1*, *cyp26b1* and *cyp26c1* in adult gonads. In adult testes (n=3), *aldh1a2* expression was detected in somatic cells surrounding cysts in a localization expected for Sertoli cells (A) and *cyp26a1* expression was detected in a subset of cells in a localization expected for Leydig cells (B). The presence of germ cells expressing *cyp26a1* could not be discarded. Expression of *cyp26b1* (C) and *cyp26c1* (D) was not detected in adult testes. In adult ovaries (n=2), *aldh1a2* was detected in somatic cells but not in the oocytes (E,I). Expression of *cyp26a1* was restricted to the ooplasm of oocytes and was not detected in somatic cells (F,J). In oocytes, *cyp26a1* expression varied according to the stage of meiosis: it was barely detectable in early stage IB oocytes (*e*IB, yellow arrowhead in F, prior to the diplotene stage of meiosis), was up-regulated in late stage IB oocytes that entered meiotic arrest at diplotene stage (*l*IB, green arrowhead in F), was maintained in stage II (red arrowhead in F) and stage III oocytes (green arrowhead in J) and was not detected in stage IV oocytes (J) coinciding with the resumption of meiosis I. Expression of *cyp26b1* was detected solely in a subset of cells in the somatic tissue surrounding the oocytes (arrows in G, K) and no expression of *cyp26c1* was detected in the ovary (H,L). Scale bar: 0.1mm.

To learn if RA plays a role in gonads beyond the time of sexual fate determination and maturation, we analyzed the expression patterns of genes encoding RA-metabolic machinery in mature gonads of 6-month old adult male and female zebrafish (Figure 7A–L).

In mature testes, *aldh1a2* expression localized to cells surrounding cysts in a position characteristic of Sertoli cells (Figure 7A). Expression of *cyp26a1* in the testis was restricted to a subset of cells, probably Leydig cells according to their localization, although expression in a small subset of germ cells (i.e. germ line stem cells) cannot be discarded (Figure 7B). Expression of *cyp26b1* (Figure 7C) and *cyp26c1* (Figure 7D) was not detected in mature zebrafish testes.

In mature ovaries, *aldh1a2* expression was restricted to somatic cells surrounding oocytes, and no expression was detected in oocytes themselves (Figure 7E,I). Reciprocally, *cyp26a1* expression was not detected in somatic cells, but appeared to be restricted to the ooplasm of oocytes (Figure 7F,J). Likewise in 41-dpf adults (Figure 4G’), ovaries expressed *cyp26a1* at a barely detectable level in early stage IB oocytes (*e*IB, yellow arrowhead Figure 7F), but then strongly up-regulated expression in late stage IB oocytes (*l*IB, green arrowhead Figure 7F), which enter meiotic arrest in diplotene; oocytes then maintained this high level until stage III (green arrowhead in Figure 7J). Interestingly, expression of *cyp26a1* down-regulated in oocytes at stage IV (Figure 7J), coincident with the resumption of meiosis. Note that, although the expression of *cyp26b1* had not been observed in developing ovaries (Figure 4), in mature adult ovaries, a subset of somatic cells did express *cyp26b1* in a punctuate pattern (Figure 7G,K), which suggests the possibility that *cyp26b1* might be involved in adult ovary homeostasis. Expression of *cyp26c1*, however, was not detected at any time in ovaries (Figure 7H,L), suggesting that while this zebrafish paralog may play a role in other organs –for example, in the retina (Figure 3A)-, it does not act in gonads.

### 6. Expression analysis of genes encoding the RA-metabolic machinery during the onset of meiosis

To learn whether the sexually dimorphic expression pattern of zebrafish *cyp26a1* correlates with the onset of meiosis, we compared the expression pattern of *cyp26a1* to that of zebrafish orthologs of tetrapod meiosis markers (reviewed in 18). In mouse, the RA-producing duct and tubules of the mesonephros are initially connected to the anterior of the bipotential gonad, and as ovaries down-regulate *Cyp26b1*, the RA target gene *Stra8* (*Stimulated by Retinoic Acid gene 8*) [78–80], which is required for the initiation of meiosis and thereby serves as a pre-meiotic marker [24,39,44,45,81], experiences an anterior–posterior wave of up-regulation accompanied by the down-regulation of the pre-meiotic pluripotent cell marker *Pou5f1*(*Oct4*) in germ cells entering meiosis and the up-regulation of the early meiotic marker *Sycp3* ( *Synaptonemalcomplex* *protein 3*) [82], which is required to assemble the synaptonemal complex during meiotic prophase [83].

To our surprise, *in silico* screening of the zv9 version of the zebrafish genome database [15] by BLAST analysis using either mouse or human STRA8 protein sequence did not return any sequences with significant similarity (e-value<1). Likewise, a survey of four other teleost reference genomes (stickleback, Tetraodon, fugu, medaka) as well as the basally diverging spotted gar genome did not identify *Stra8*. We did, however, find an ortholog for *Stra8* (fgenesh2_pg.scaffold_222000016, reciprocal best blast hit e-value of 8e^-09^ vs. human) in the genome of the basally diverging chordate amphioxus *Branchiostoma* *floridae* , and in the elephant shark [84], supporting the hypothesis that *stra8* was present in stem chordates and stem gnathostomes, but was lost secondarily in the ray fin fish lineage [84] at least prior to the divergence of spotted gar before the TGD [85]. Future analysis of Stra8 in cartilaginous fish is needed to test if its ancestral function was related to the initiation of meiosis, or alternatively, if Stra8 function in meiosis was a tetrapod innovation. In any case, the absence of *Stra8* in teleosts suggests that if RA plays a role in the regulation of the initiation of meiosis in teleosts, it does not act through Stra8 as it does in mammals [81].

**Figure 8:**
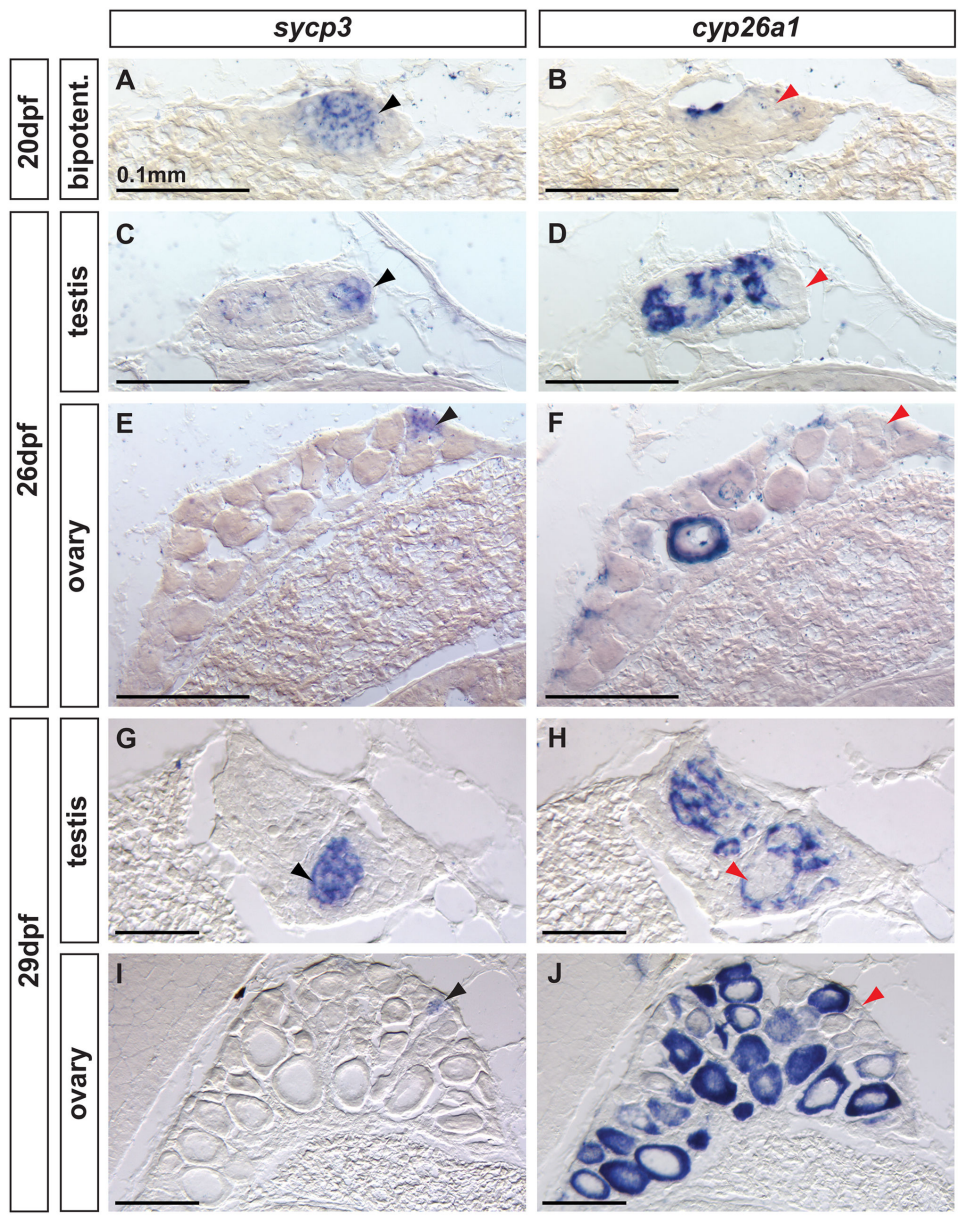
Complementary expression of the meiotic recombination marker *sycp3* and *cyp26a1* in developing gonads. In bipotential gonads at 20 dpf (A,B: n=8), germ cells expressed the meiotic recombination marker *sycp3* (black arrowhead in A) in a non-overlapping pattern with *cyp26a1* expression, which was mostly restricted to the dorsal part of the gonad (revealing that *sycp3-*expressing cells did not express *cyp26a1* (red arrowhead in B). Expression of the meiotic marker *sycp3* was detected in bipotential gonads of all animals analyzed (A, n=8) suggesting that some germ cells entered meiosis in all juveniles regardless of their definitive sex. In differentiating testes at 26 dpf (C,D: n=2) and 29 dpf (G, H: n=2), islands of germ cells that expressed *sycp3* (black arrowheads in C, G) were found in an area in which RA was likely not degraded due to lack of *cyp26a1* expression (red arrowheads in D,H). In contrast, in differentiating ovaries at 26 dpf (E, F: n=2) and 29 dpf (I, J: n=2), *sycp3* was expressed in small germ cells (black arrowheads in E, I) that did not express *cyp26a1* (red arrowheads in F, J). The expression of *cyp26a1* was restricted to the ooplasm of oocytes that reached diplotene stage and entered in meiotic arrest (F,J). Scale bar: 0.1mm.

Analysis of the early meiotic marker *Sycp3* by in situ hybridization in adjacent sections showed that all zebrafish analyzed at the bipotential stage (20 dpf, n=8) showed germ cells expressing *sycp3* throughout the gonad, while cells expressing *cyp26a1* were mostly restricted to the dorsal surface (Figure 8A,B and Figure 4C). Transitioning and immature gonads (26 dpf and 29 dpf) expressed *sycp3* and *cyp26a1* in a complementary, non-overlapping fashion. In males, germ cells expressing *sycp3* were located in specific regions (black arrowhead in Figure 8C, G) in which no somatic *cyp26a1* expression was observed (red arrowhead in Figure 8D, H) as predicted by the hypothesis that Cyp26a1 inhibits meiosis. In females, *sycp3* expression was observed only in oocytes (black arrowhead in Figure 8E, I) that had not yet reached late stage IB, and no somatic *cyp261a1* expression was observed. Interestingly, females showed *cyp26a1* expression in the ooplasm of late stage IB oocytes (Figure 8F,J), but not in early oocytes that expressed *sycp3* (Figure 8E,I), revealing a complementary non-overlapping expression pattern of *sycp3* and *cyp26a1* during oocyte maturation, as would be expected if Cyp26a1 inhibited meiosis.

**Figure 9:**
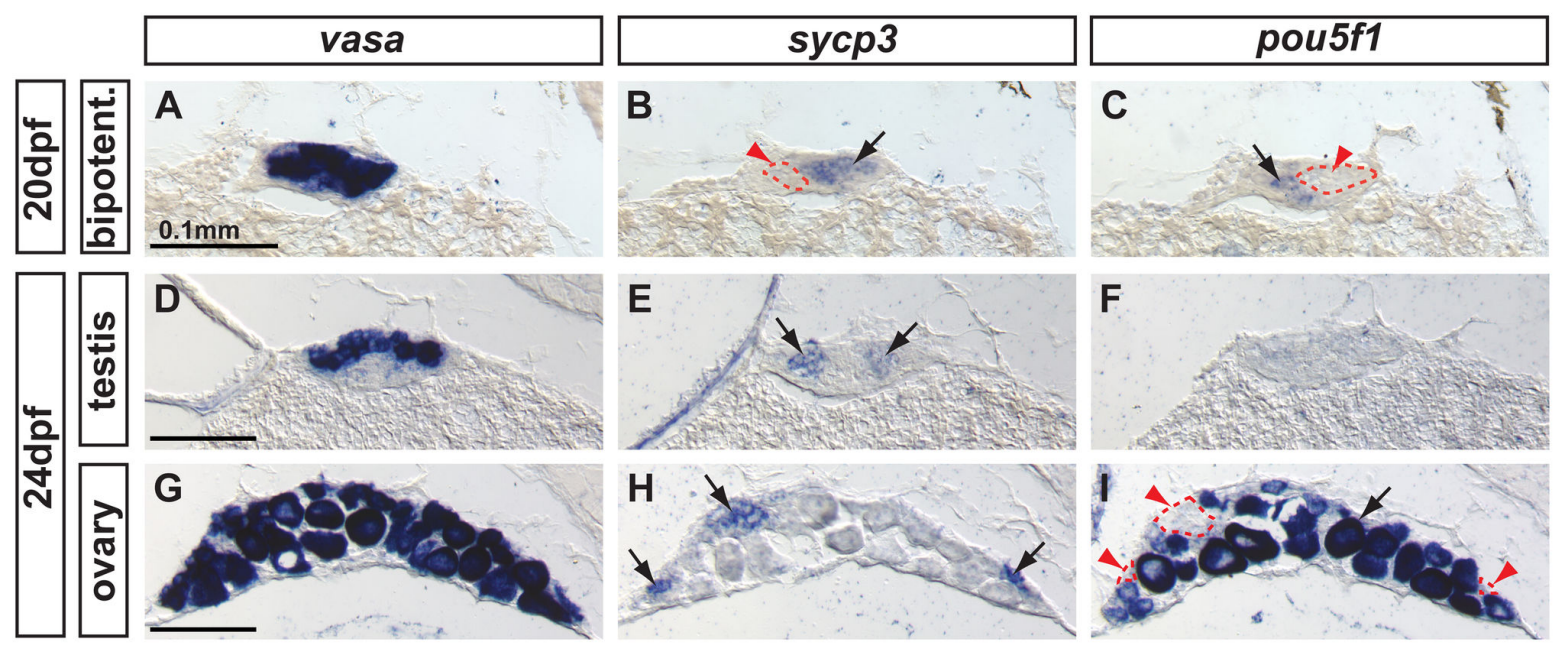
Complementary expression of the meiotic recombination marker *sycp3* and the pluripotent marker *pou5f1*(oct4) in developing gonads. In bipotential gonads at 20 dpf, comparison of the expression of the germ cell marker *vasa* (A), the synaptonemal complex marker *sycp3* (arrow in B) and the pluripotent gene *pou5f1* (arrow in C) revealed that *sycp3* and *pou5f1* were both expressed in germ cells but in a complementary non-overlapping fashion (red arrowheads and dashed lines in B and C). In differentiating testes at 24 dpf, expression of *vasa* was detected in germ cells (D) revealing that only some of the germ cells expressed *sycp3* (arrows in E) but none of them expressed *pou5f1* (F). In differentiating ovaries at 24 dpf, *vasa* labeled germ cells (G), and *sycp3* only labeled those germ cells that were small (arrows in H), which interestingly did not express *pou5f1* (red arrowheads and dashed lines in I) and had not reached the late stage IB. Complementarily, the larger oocytes that had reached diplotene stage expressed *pou5f1* (arrow in I). Scale bar indicated per each raw: 0.1mm.

Finally, the expression of the pluripotent marker *pou5f1* and the early-meiotic marker *sycp3* in bipotential gonads at 20 dpf showed non-overlapping expression domains in groups of germ cells, as shown by *vasa* expression (Figure 9A–C). This complementary pattern is expected if pre-meiotic germ cells down-regulate *pou5f1* as they enter meiosis and begin to express *sycp3* [18]. Immature testes at 24 dpf showed groups of germ cells expressing *sycp3*, but no cells expressing *pou5f1* (Figure 9 E, F), which suggests the presence of abundant cysts of germ cells undergoing meiosis, but not obvious pre-meiotic spermatogenic cells at this stage. Immature ovaries at 24 dpf contained oocytes at early stage IB progressing through meiosis and expressing *sycp3* but not *pou5f1* (Figure 9G–I red arrowheads). Interestingly, oocytes at late stage IB resumed *pou5f1* expression (Figure 9G–I black arrow), which is compatible with the proposed role of Pou5f1 in promoting primordial germ cell survival by preventing apoptosis [86], a mechanism that has been shown to be central in late stage IB oocytes for tipping the sexual fate of the gonad towards the female pathway in zebrafish [5,8,87].

## Discussion

This work provides, to our knowledge, the first comprehensive genomic and molecular analysis of the genetic machinery that regulates the synthesis and degradation of RA at the time that zebrafish gonads tip their sexual fate towards the male or female pathway. Our findings reveal several significant differences between RA-regulated gonadogenesis in zebrafish and tetrapods, including which cells express RA-synthesizing enzymes, which paralog encodes gonadal RA-degrading enzymes, whether RA-degrading enzymes are expressed in a dimorphic fashion, whether Stra8 regulates entry into meiosis, and whether the onset of meiosis is sexually dimorphic.

### 1. *During the critical time window for gonadal sex determination, aldh1a2, which encodes an RA-synthesizing enzyme, is expressed in the gonad in zebrafish rather than in the mesonephros as in mouse*

**Figure 10:**
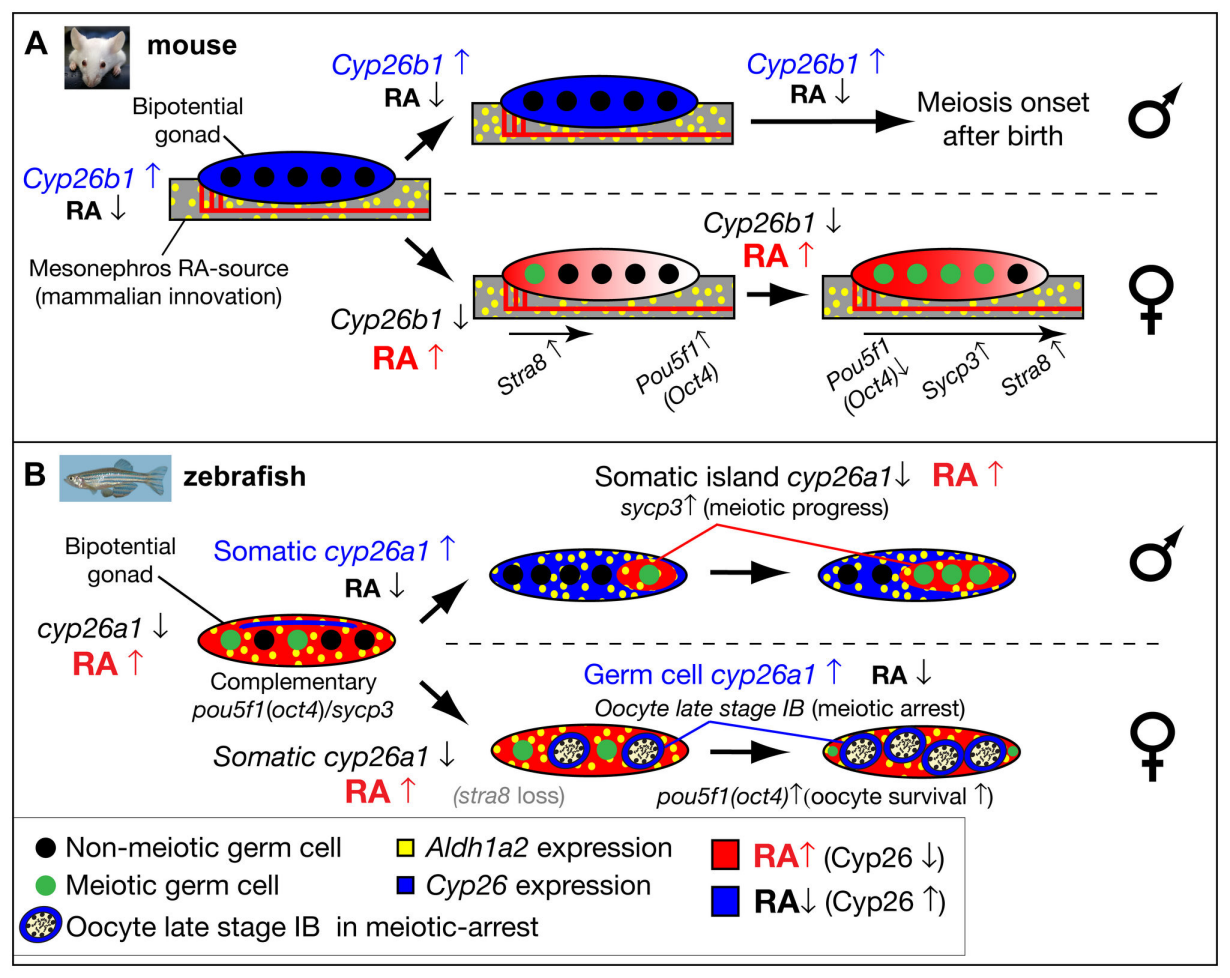
Model for the role of retinoic acid and meiotic progression during gonadogenesis in mouse and zebrafish. In mouse (A), *Aldh1a2* (yellow) in the mesonephros provides the RA-source that regulates gonad development (reviewed in [18]), while in zebrafish (B), *aldh1a2* is expressed by somatic cells within the gonad (yellow), and thereby provides an internal RA-source. In mouse, non-meiotic germ cells (black circles) in bipotential gonads are protected from RA by high expression of *Cyp26b1* (blue), while in zebrafish, Cyp26a1 expression (blue) is restricted to cells at the dorsal surface near the body cavity, and thereby germ cells elsewhere in the gonad are not protected from RA (red) and they are able to enter into meiosis (green circles). In mouse, sexually dimorphic expression of *Cyp26b1* causes low levels of RA (blue) in testes (males, top in A) and high levels of RA (red) that diffuses from the mesonephros to the ovaries in an anterior to posterior wave (females, bottom in A), which results in a sexually dimorphic onset of meiosis. The onset of meiosis in mouse follows an anterior–posterior wave, accompanied by up-regulation of *Stra8*, and *Sycp3* and down-regulation of *Pou5f1*(Oct4) [18]. In zebrafish, the sexually dimorphic expression of *cyp26a1* differs in time and location from those of *Cyp26b1* in mouse. In zebrafish males (top in B), somatic cells up-regulate *cyp26a1*, and meiotic cells expressing *sycp3* localize to somatic islands (red) that lack *cyp26a1* expression. This model is consistent with a role for Cyp26a1 in degrading RA and thereby protecting nearby germ cells from progressing through meiosis. Consistent with this model, in females (bottom in B), *cyp26a1* is not expressed in somatic cells, but it is up-regulated in the ooplasm (blue) of oocytes at late stage IB that have reached diplotene stage and have entered in meiotic arrest, and also express *pou5f1*(oct4). The co-expression of *cyp26a1* and *pou5f1*(oct4) is compatible with the proposed roles of these genes in mouse on promoting germ cell survival and preventing apoptosis [41,86], mechanisms that have been shown to be central for tipping the sexual fate of the gonad toward the female pathway in zebrafish [5,8,87].

In mouse, *Aldh1a2* is not expressed in the gonad as it is in zebrafish, but is strongly expressed in the adjacent mesonephros at the bipotential stage, leading to the hypothesis that mesonephros-derived RA provides a regulatory system that drives an anterior–posterior wave of meiotic progression in the fetal ovary [23] (Figure 10A). In contrast to mouse, zebrafish gonadal somatic cells express *aldh1a2* throughout gonadogenesis, suggesting that the gonad can provide a continuing source of RA that controls the development of the germline (Figure 7B). This model is consistent with the fact that in zebrafish, the mesonephros lies distant from the gonad and does not contact the gonad during the critical time window of sex determination as it does in mouse. In chicken, as in zebrafish, *Aldh1a2* is expressed in the embryonic gonad [47,49], which suggests by parsimony that the expression of *Aldh1a2* in the gonads is likely the ancestral condition in vertebrates and that the lack of *aldh1a2* expression in the mouse gonad is probably an evolutionary innovation in the mammalian lineage (Figure 10A). Somatic cells in mouse testis begin to express *Aldh1a1* shortly after the onset of *Sry* expression, which implies a local source of RA in the testis in addition to the Aldh1a2-derived RA from the mesonephros [46]. Male-specific *Aldh1a1* expression in gonads may provide low levels of RA that might facilitate early events in testes development or may prefigure later postnatal spermatogenesis, rather than functioning in the dimorphic induction of the onset of meiosis [46]. In zebrafish, it is likely that the secondary loss of the *aldh1a1* gene, a paralog of *aldh1a2*, which occurred during the evolution of the teleost lineage [56], was not lethal or deleterious because its function in the gonad was redundantly covered by *aldh1a2*. The loss of *aldh1a1* in teleosts, however, might have decreased the evolvability of *aldh1a2*, which became the only source of RA during gonad development, given our evidence that *aldh1a3* is not expressed in the zebrafish gonad.

### 2. *Independent partitioning of gonadal subfunctions between Cyp26a1 and Cyp26b1 paralogs in teleosts and tetrapods*

Our study revealed that zebrafish and mouse express different *Cyp26* paralogs during early gonad development; mainly *Cyp26a1* in zebrafish and mainly *Cyp26b1* in mouse. Our phylogenetic analysis and study of conserved syntenies between zebrafish, medaka, and mouse support the proposed gene nomenclature [63] and show that orthologies are correctly assigned.

We propose that the ancestral vertebrate Cyp26 pro-ortholog was already responsible for the regulation of RA action during gonadogenesis, and that after expansion of the *Cyp26* family in stem vertebrates [54,88], independent subfunction partitioning events [72,73] led to different paralogs — *cyp26a1* and *Cyp26b1*- functioning as the main players of RA degradation in the gonads of zebrafish and tetrapods, respectively. Comparative analysis of the three *cyp26* paralogs during late eye development in zebrafish (Figure 3) and mouse [74] suggests that the independent subfunction partitioning we found in gonads does not necessarily extend to other organs. The modular fashion in which gonadal and retinal subfunctions partitioned in teleosts and tetrapods predicts that independent transcriptional regulatory elements control each subfunction.

Interestingly, a recent work detected *cyp26b1* transcript by RT-PCR in 100-day post hatching gonads of Japanese flounder males induced by high temperature [89]. No data, however, is available concerning the cellular distribution of *cyp26b1* expression in flounder, nor whether *cyp26a1* and *cyp26c1* are expressed in flounder gonads. Future analysis of all three *cyp26* paralogs in the Japanese flounder and other teleosts, together with a comparative analysis of the regulatory regions of the *Cyp26* paralogs of teleosts and tetrapods will clarify the evolution of cyp26 subfunctions during the evolution of teleosts and tetrapods.

3. Expression of *cyp26a1* in zebrafish does not suggest a role in the dimorphic onset of meiosis as in tetrapods, but correlates with a role in the differential progression of meiotic oocytes in both males and females

In mammals, birds, and amphibians, the sexually dimorphic expression of *Cyp26b1* appears to regulate the timing of meiotic onset, which differs in oocytes and spermatocytes [18,23,24,47,48]. In early mouse gonads (11.5 dpc), males and females both express *Cyp26b1* throughout the gonad, but after the expression of *Sry* and gonads begin sexual differentiation (12.5 dpc), *Cyp26b1* expression up-regulates in males but down-regulates in females [23,24] (Figure 9A). In male mice, up-regulation of *Cyp26b1* in gonadal somatic cells is at a time and place appropriate to protect germ cells from RA, and thereby to prevent oogenesis in males by retarding *Stra8* expression and postponing the onset of meiosis until postnatal stages [23,24] (Figure 9A). In female mice, the absence of Cyp26b1 leads to the activation of *Stra8* in germ cells, and promotes the onset of meiosis and oocyte development, thereby reinforcing the female pathway (Figure 9A).

In mouse females, it has been hypothesized that the presence of germ cells committed to meiosis reinforces ovarian fate by antagonizing the testis pathway [22]. We wondered if this hypothesis also applied to zebrafish, and so tested whether some of zebrafish juveniles (presumably females) have an early onset of meiosis that could reinforce the ovarian fate of the gonad, while others (presumably males) have a delayed onset of meiosis and develop testes. Our results reveal that the meiotic marker *sycp3* is expressed in bipotential gonads of all juvenile zebrafish analyzed, suggesting that, in contrast to mammals, the onset of meiosis in zebrafish gonads is not sexually dimorphic and therefore the onset of meiosis does not appear to be a cue that biases gonads towards the female or male pathway (Figure 8). Although Sycp3 is an essential component of the synaptonemal complex in meiotic cells [40] and is generally used as a meiotic marker [23,90], we cannot verify that all *sycp3* expressing cells in zebrafish are progressing through meiosis [91].

Our expression analyses comparing genes that encode enzymes that regulate RA synthesis and degradation markers for somatic and germ cells and markers for meiotic and pluripotent cells are compatible with a model in which RA — or, less likely [28] a non-RA Cyp26-digested molecule [29] — is involved in the progression of meiosis in zebrafish (Figure 10). First, all juveniles with bipotential gonads containing *sycp3-*expressing meiotic germ cells express *aldh1a2* broadly throughout the gonad but express *cyp26a1* in a few peripheral cells, suggesting that most germ cells, including those randomly scattered pre-meiotic germline stem cells labeled by *nanos2* [92], are developing in a cellular environment that according to gene expression patterns should contain RA (Figure 9B). Second, the result that germ cells expressing the meiotic cell marker *sycp3* appear only at a substantial distance from cells expressing *cyp26a1* in the same gonad suggests that the *cyp26a1-*expressing cells inhibit meiosis. Third, the finding that the onset and maintenance of *aldh1a2* and *cyp26a1* expression occurs in gonads containing no germ cells suggests that in bipotential zebrafish gonads, the gonadal soma rather than the germ line regulates RA and the onset of meiosis.

These considerations lead us to suggest the hypothesis that the continuous availability of RA to germ cells in bipotential gonads prohibits the sexually dimorphic onset of meiosis in zebrafish and is consistent with the initial development of meiotic oocytes in all juvenile zebrafish (Figure 10B). This model contrasts with mouse, in which the female-specific down-regulation of *Cyp26b1* triggers the onset of meiosis, while the male-specific up-regulation of *Cyp26b1* delays the onset of meiosis until after birth (Figure 10A). Our model for zebrafish is compatible with the teleost-specific loss of *stra8*, the “meiotic gatekeeper” [39] gene that in tetrapods mediates sexually dimorphic onset of meiosis (Figure 10B).

Interestingly, we observed that zebrafish express *cyp26a1* dimorphically in gonads committed to the male or female pathway, consistent with a role of RA regulating meiotic progression during spermatogenesis and oogenesis (Figure 9B). In zebrafish males, the localized presence of *sycp3*-positive germ cells entering meiosis only where associated with somatic cells free of *cyp26a1* expression suggests the hypothesis that cells expressing *cyp26a1* degrade RA and thus prevent nearby germ cells from entering RA-induced meiosis. This hypothesis is consistent with a model in which RA availability is important for the progression of meiosis, and Cyp26a1 acts as a meiosis-inhibiting factor [23]. In females, *cyp26a1* expression up-regulates in oocytes that have completed recombination (late stage IB) and are entering meiotic arrest in diplotene. This observation, together with the fact that *cyp26a1* expression disappears late in oocyte development at stage IV when meiosis resumes, is compatible with the Cyp26a1-mediated degradation of RA in oocytes that prevents the progression of meiosis and maintains meiotic arrest. This finding is consistent with results in mouse, in which RA prevents meiotic arrest in testes of 13.5 dpc embryos [93]. Interestingly, in zebrafish developing gonads, oocytes entering meiotic arrest at diplotene also express both the meiosis-inhibiting gene *cyp26a1* and the pluripotent marker *pou5f1* (*oct4*). The convergence of *cyp26a1* and *pou5f1* expression in zebrafish oocytes at meiotic arrest is compatible with the proposed roles of *Pou5f1* and *Cyp26b1* in mouse germ cells in promoting survival and preventing apoptosis [41,86], mechanisms that tip the sexual fate of the gonad toward the female pathway in zebrafish [5,8,87].

Overall, this work provides the knowledge base on RA metabolic gene expression necessary for the design of experiments that alter RA signaling in zebrafish developing gonads to test the model stemming from our results for the role of RA signaling in the progress of meiosis. In addition, further work is required to understand the relationship of RA signaling to the Fgf9/Wnt4 seesaw that helps regulate gonad fate in mouse [90,94–98]. Especially intriguing with respect to the evolution of sex determination is the lack of an *fgf9* gene in zebrafish and other teleosts [99,100] and the presence of two *wnt4* genes [101], genomic variations with interesting consequences for zebrafish sex determination.

## Materials and Methods

### 1. Animals and Ethics Statement

AB strain zebrafish were used in all experiments. Animals were reared and collected under standard conditions and were handled in accordance with good animal practice. The University of Oregon Institutional Animal Care and Use Committee approved all animal work (Animal Welfare Assurance Number A-3009-01, IACUC protocol 08-13).

### 2. *dead end* morpholino injections

To obtain animals lacking germ cells, wild-type zebrafish embryos were injected at the 1-2 cell stage with antisense morpholino oligonucleotide (Gene Tools, Oregon) directed against *dead end* as described [77]. Sibling non-injected embryos and a fraction of *dnd* MO-injected embryos were fixed at 24 hours post-fertilization to confirm the presence or absence of germ cells by whole-mount in situ hybridization using *vasa* probe as described [75].

### 3. *In situ* hybridization

*In situ* hybridization experiments on zebrafish cryosections were performed as described [59]. Adjacent sections of gonads were obtained by placing five consecutive 16-μm sections of the gonad on five different slides. Generally, between two to nine animals at each stage were analyzed per each probe, as indicated in each figure legend. Probes for *aldh1a2* and *aldh1a3* were made as described [56]; *amh* probe was made as described and used as a somatic cell marker to distinguish differentiating testes and ovaries when morphological features were not clear [59]; and probe for *vasa* was made from its 3’ end as described [75]. Fragments from cDNA of *cyp26a1* (nucleotides 507-1848 of NM_131146), *cyp26b1* (nucleotides 518-1548 of NM_212666), *cyp26c1* (nucleotides 198-1533 of NM_001029951), *pou5f1* (*oct4*) (nucleotides 705-1482 of NM_131112), and *sycp3* (nucleotides 265-884 of NM_001040350) were cloned in TOPO vector (Invitrogen) and used to synthesize DIG-labeled riboprobes (Boehringer Mannheim). The protocol for the three-color fluorescent in situ hybridization is described in wiki.zfin.org/display/prot/3+color+Fluorescent+in+situ+on+sections.

### 4. Phylogenetic Tree and Analysis of synteny conservation

An alignment of Cyp26 proteins from vertebrates and cephalochordates was generated with clustalX [102]. This alignment was used to generate phylogenetic trees inferred by Neighbor-Joining using the MEGA2 package [103] and Maximum likelihood (ML) using PhyML [104] following a LG+I+G model, the alpha parameter of the gamma distribution and a proportion of invariable sites were estimated from the sample, and four categories of substitution rates were taken into account. The topology, branch lengths, and rate parameters of the tree were optimized. Tree topologies obtained by ML and NJ were identical. Confidence of tree topologies (NJ and ML) was inferred by 100 replications to calculate bootstrap values supporting each node of the tree, and differences between the two methods were minor. Cyp26 proteins from cephalochordates [54], which diverged at the base of chordate phylogeny, were used as the outgroup to root the tree. Genomic database accession numbers from NCBI (www.ncbi.nlm.nih.gov), Ensembl (www.ensembl.org) and JGI (www.jgi.doe.gov): Tetrapods: *Homo sapiens* (Hsa CYP26A1, NP_000774.2; CYP26B1, NP_063938.1; CYP26C1, NP_899230.2); *Mus musculus* (Mmu: cyp26a1, NP_031837.1; cyp26b1, NP_780684.1; cyp26c1, NP_001098671.1); *Gallus gallus* (Gga: Cyp26a1, NP_001001129.1; Cyp26b1, XP_426366.2; Cyp26c1, XP_421678.2); Teleosts: *Danio rerio* (Dre: cyp26a1, NP_571221.2; cyp26b1, NP_997831.1; cyp26c1, NP_001025122.2); *Takifugu* *rubripes* (Tru: Cyp26a1, ENSTRUG00000002005; Cyp26b1, ENSTRUG00000012613; Cyp26c1, ENSTRUG00000012794); *Gasterosteus* *aculeatus* (Gac: Cyp26a1, ENSGACG00000015370; Cyp26b1, ENSGACG00000018809; Cyp26c1, ENSGACG00000011080); *Oryzias latipes* (Ola: Cyp26a1, ENSORLG00000014516; Cyp26b1, ENSORLG00000003465; Cyp26c1, ENSORLG00000002036); Cephalochordates: *Branchiostoma* *floridae* (Cyp26a, Bfl Brafl1:87630; Cyp26b, Brafl1|124944 as in JGI inferred by [54]). Circleplots graphically represent user-selected chromosomes as arcs along the circumference of a circle. The origins of lines connecting positions along the arcs represent pairs of orthologous genes between two different species. Relationships of orthology and plots were generated by the Synteny Database (version Ens56; http://syntenydb.uoregon.edu/synteny_db/ [69]). Each genomic neighborhood consists of a 5Mb window centered on each Cyp26 gene: *CYP26B1* in Mmu6 (82,022-87,022 kb); *CYP26A1* and *CYP26C1* in Mmu19 (35,260-40,272 Mb); *cyp26a1* in Ola19 (19,025-24,025 Kb); *cyp26b1* in Ola18 (2,121-7,121 Kb); and *cyp26c1* in Ola15 (6,956-11,956 Kb). Gene loci that are close to each other may appear to overlap as a single connecting line in circle-plots due to the selected graph resolution, and lines based on best reciprocal blast hits that were not significantly different were not considered. Clusters of conserved synteny were created by coupling results from the reciprocal best hit BLAST pipeline with the use of a 100-gene sliding-window analysis that links chromosome segments with conserved synteny (for details see 69). Clusters that link chromosomal segments between different species represent orthologous syntenic conservation (e.g. Figure 2C–E), and clusters that link chromosomal segments within the same species represent paralogous syntenic conservation (e.g. Figure 2F).

## Supporting Information

Figure S1 **Cyp26 orthologies between zebrafish and medaka were supported by clusters of conserved synteny, which extends conclusions from zebrafish to other teleost models.** A: *cyp26a1* in *Dre*12 and *Ola*19; B: *cyp26b1* in *Dre*7 and *Ola*18; and *cyp26c1* in *Dre*17 and *Ola*15. *Cyp26* orthologs have been labeled with larger fonts, and names of gene neighbors can be surfed in the high-resolution pdf electronic files.
(PDF)

Figure S2 **Comparative genomic analysis of synteny conservation between mouse and medaka supports conclusions from comparison of zebrafish and mouse (Figure 2), ruling out the possibility of reciprocal gene loss in different lineages, and supports the notion that teleost *cyp26a1* and tetrapod *cyp26b1* are not orthologs.** B: *cyp26b1* in *Mmu*6 and *Ola*18; B: *Cyp26a1* in *Mmu*19 and *Ola*19; and *Cyp26c1* in *Mmu*19 and *Ola*15. Large fonts label *cyp26* orthologs and names of gene neighbors are legible in the high-resolution pdf electronic files.
(PDF)
