# Supplementary material for: Retinoic Acid Metabolic Genes, Meiosis, and Gonadal Sex Differentiation in Zebrafish
Source: PLoS One. 2013 Sep 10;8(9):e73951. doi: 10.1371/journal.pone.0073951 (PMC3769385; doi:10.1371/journal.pone.0073951)

Additional Figure S1. Cyp26 orthologies between zebrafish and medaka were supported by clusters of conserved synteny, which extends conclusions from zebrafish to other teleost models. A: *cyp26a1* in *Dre*12 and *Ola*19; B: *cyp26b1* in *Dre*7 and *Ola*18; and *cyp26c1* in *Dre*17 and *Ola*15. *Cyp26* orthologs have been labeled with larger fonts, and names of gene neighbors can be surfed in the high-resolution pdf electronic file.

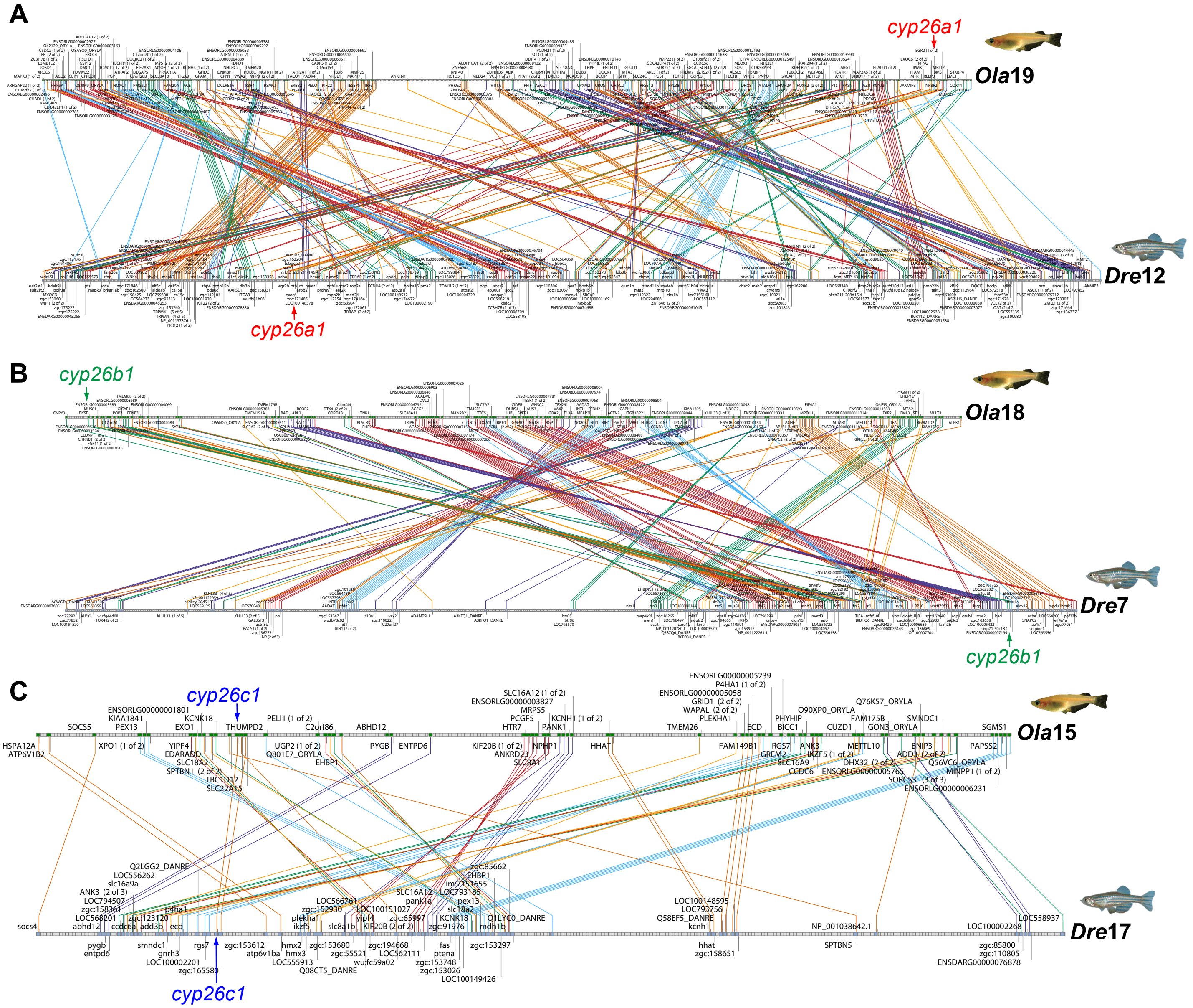

Supplement: Figure S1 — Cyp26 orthologies between zebrafish and medaka were supported by clusters of conserved synteny, which extends conclusions from zebrafish to other teleost models. A: cyp26a1 in Dre12 and Ola19; B: cyp26b1 in Dre7 and Ola18; and cyp26c1 in Dre17 and Ola15. Cyp26 orthologs have been labeled with larger fonts, and names of gene neighbors can be surfed in the high-resolution pdf electronic files. (PDF) [file pone.0073951.s001.pdf]
