# Supplementary material for: Retinoic Acid Metabolic Genes, Meiosis, and Gonadal Sex Differentiation in Zebrafish
Source: PLoS One. 2013 Sep 10;8(9):e73951. doi: 10.1371/journal.pone.0073951 (PMC3769385; doi:10.1371/journal.pone.0073951)

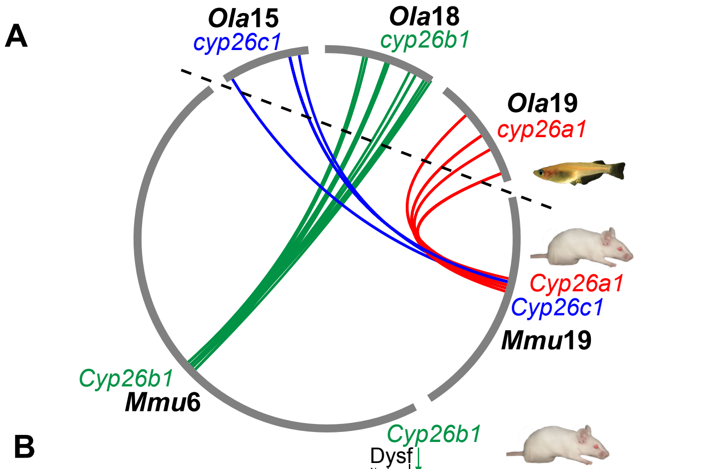

Additional Figure S2. Comparative genomic analysis of synteny conservation between mouse and medaka supports conclusions from comparison of zebrafish and mouse (Fig. 2), ruling out the possibility of reciprocal gene loss in different lineages, and supports the notion that teleost *cyp26a1* and tetrapod *cyp26b1* are not orthologs. B: *cyp26b1* in *Mmu*6 and *Ola*18; B: *Cyp26a1* in *Mmu*19 and *Ola*19; and *Cyp26c1* in *Mmu*19 and *Ola*15. Large fonts label *cyp26* orthologs and names of gene neighbors are legible in the high-resolution pdf electronic file.

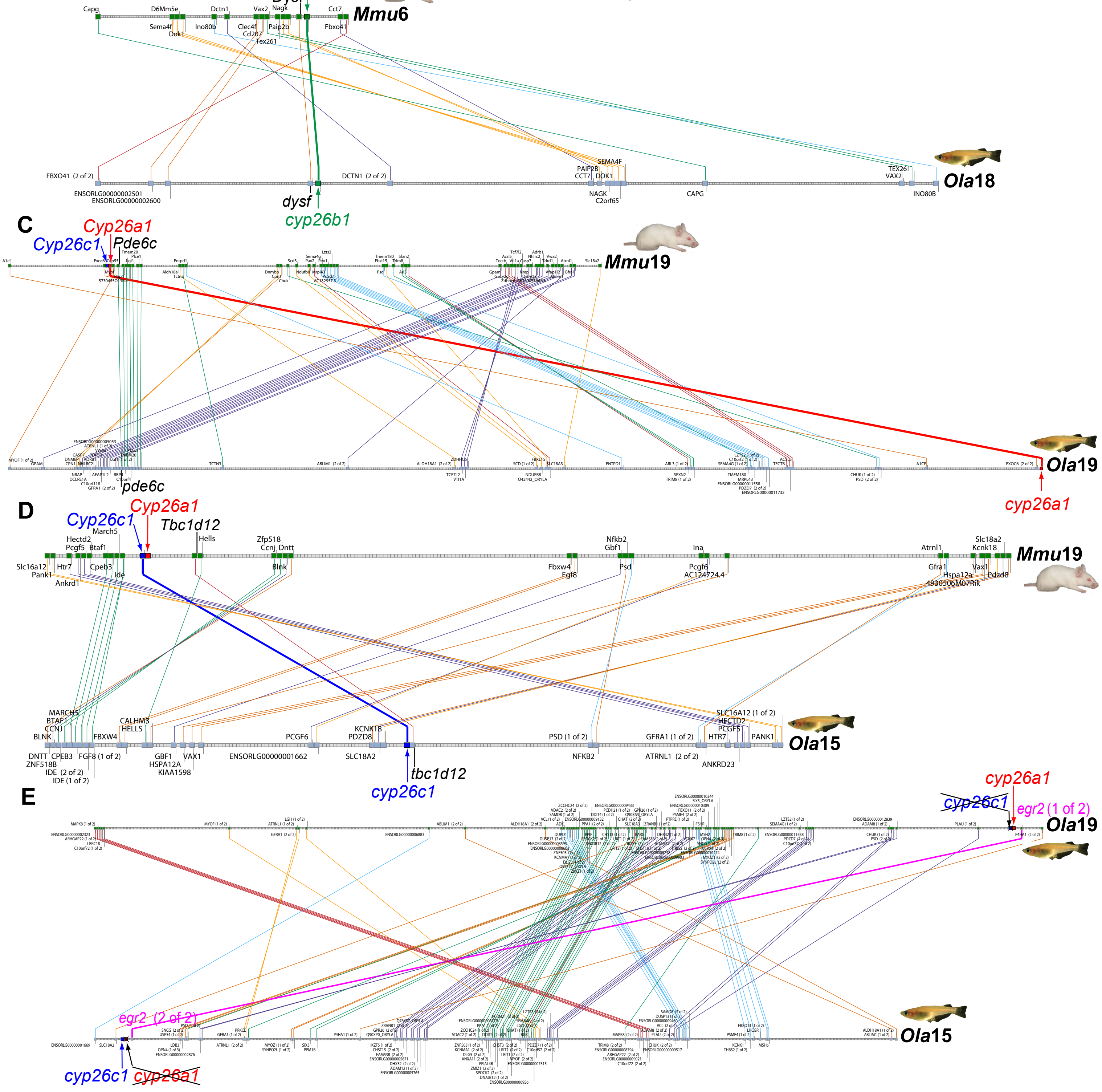

Supplement: Figure S2 — Comparative genomic analysis of synteny conservation between mouse and medaka supports conclusions from comparison of zebrafish and mouse (Figure 2), ruling out the possibility of reciprocal gene loss in different lineages, and supports the notion that teleost cyp26a1 and tetrapod cyp26b1 are not orthologs. B: cyp26b1 in Mmu6 and Ola18; B: Cyp26a1 in Mmu19 and Ola19; and Cyp26c1 in Mmu19 and Ola15. Large fonts label cyp26 orthologs and names of gene neighbors are legible in the high-resolution pdf electronic files. (PDF) [file pone.0073951.s002.pdf]
